# Building a transdisciplinary expert consensus on the cognitive drivers of performance under pressure: An international multi-panel Delphi study

**DOI:** 10.3389/fpsyg.2022.1017675

**Published:** 2023-01-18

**Authors:** Lucy Albertella, Rebecca Kirkham, Amy B. Adler, John Crampton, Sean P. A. Drummond, Gerard J. Fogarty, James J. Gross, Leonard Zaichkowsky, Judith P. Andersen, Paul T. Bartone, Danny Boga, Jeffrey W. Bond, Tad T. Brunyé, Mark J. Campbell, Liliana G. Ciobanu, Scott R. Clark, Monique F. Crane, Arne Dietrich, Tracy J. Doty, James E. Driskell, Ivar Fahsing, Stephen M. Fiore, Rhona Flin, Joachim Funke, Justine M. Gatt, P. A. Hancock, Craig Harper, Andrew Heathcote, Kristin J. Heaton, Werner F. Helsen, Erika K. Hussey, Robin C. Jackson, Sangeet Khemlani, William D. S. Killgore, Sabina Kleitman, Andrew M. Lane, Shayne Loft, Clare MacMahon, Samuele M. Marcora, Frank P. McKenna, Carla Meijen, Vanessa Moulton, Gene M. Moyle, Eugene Nalivaiko, Donna O'Connor, Dorothea O’Conor, Debra Patton, Mark D. Piccolo, Coleman Ruiz, Linda Schücker, Ron A. Smith, Sarah J. R. Smith, Chava Sobrino, Melba Stetz, Damien Stewart, Paul Taylor, Andrew J. Tucker, Haike van Stralen, Joan N. Vickers, Troy A. W Visser, Rohan Walker, Mark W. Wiggins, Andrew Mark Williams, Leonard Wong, Eugene Aidman, Murat Yücel

**Affiliations:** ^1^Turner Institute for Brain and Mental Health, School of Psychological Sciences, Monash University, Melbourne, VIC, Australia; ^2^Walter Reed Army Institute of Research, Silver Spring, MD, United States; ^3^APS College of Sport and Exercise Psychologists, Melbourne, VIC, Australia; ^4^School of Psychology and Wellbeing, University of Southern Queensland, Toowoomba, QLD, Australia; ^5^Stanford University, Stanford, CA, United States; ^6^Wheelock College of Education and Human Development, Boston University, Boston, MA, United States; ^7^Department of Psychology, University of Toronto, Toronto, ON, Canada; ^8^National Defense University, Washington, DC, United States; ^9^Australian Army Psychology Corps, Canberra, ACT, Australia; ^10^U.S. Army DEVCOM Analysis Center, Natick, MA, United States; ^11^Physical Education & Sport Sciences Department, University of Limerick, Limerick, Ireland; ^12^Adelaide Medical School, University of Adelaide, Adelaide, SA, Australia; ^13^School of Psychological Sciences, Macquarie University, Sydney, NSW, Australia; ^14^Department of Psychology, American University of Beirut, Beirut, Lebanon; ^15^Florida Maxima Corporation, Orlando, FL, United States; ^16^Norwegian Police University College, Oslo, Norway; ^17^Department of Psychology, and Institute of Simulation and Training, University of Central Florida, Orlando, FL, United States; ^18^Aberdeen Business School, Robert Gordon University, Aberdeen, United Kingdom; ^19^Department of Psychology, Heidelberg University, Heidelberg, Germany; ^20^School of Psychology, University of New South Wales, Kensington, NSW, Australia; ^21^Neuroscience Research Australia, Sydney, NSW, Australia; ^22^The University of Newcastle, Callaghan, NSW, Australia; ^23^School of Biomedical Sciences and Pharmacy, The University of Newcastle, Callaghan, NSW, Australia; ^24^US Army Research Institute of Environmental Medicine (USARIEM), Natick, MA, United States; ^25^Department of Movement Sciences, KU Leuven, Leuven, Belgium; ^26^Defense Innovation Unit, Mountain View, CA, United States; ^27^School of Sport, Exercise and Health Sciences, Loughborough University, Loughborough, United Kingdom; ^28^United States Naval Research Laboratory, Washington, DC, United States; ^29^Department of Psychiatry, University of Arizona, Tucson, AZ, United States; ^30^School of Psychology, The University of Sydney, Darlington, NSW, Australia; ^31^Sydney School of Education and Social Work, The University of Sydney, Darlington, NSW, Australia; ^32^Sport, Physical Activity Research Centre (SPARC), School of Sport, University of Wolverhampton, Wolverhampton, United Kingdom; ^33^School of Psychological Science, University of Western Australia, Perth, WA, Australia; ^34^School of Allied Health, Human Services, and Sport, La Trobe University, Melbourne, VIC, Australia; ^35^Department of Biomedical and Neuromotor Sciences, University of Bologna, Bologna, Italy; ^36^Department of Psychology, University of Reading, Reading, United Kingdom; ^37^Faculty of Sport, Allied Health and Performance Science, St Mary's University, Twickenham, United Kingdom; ^38^Mindflex Group Ltd, London, United Kingdom; ^39^Faculty of Creative Industries, Education and Social Justice, Queensland University of Technology, Brisbane, QLD, Australia; ^40^Department of Defense, Canberra, ACT, Australia; ^41^United States Department of Defense, Washington DC, United States; ^42^Fire Rescue Victoria, Melbourne, VIC, Australia; ^43^Mission Critical Team Institute, Annapolis, MD, United States; ^44^Department of Sport Psychology, Institute of Sport and Exercise Sciences, University of Münster, Münster, Germany; ^45^Consultant, Sydney, NSW, Australia; ^46^Defense Science and Technology Laboratory, Salisbury, United Kingdom; ^47^NSW Institute of Sport and Diving, Sydney, NSW, Australia; ^48^Independent Practitioner, Grand Ledge, MI, United States; ^49^Room23 Psychology, Brisbane, QLD, Australia; ^50^Altrecht Institute for Mental Health Care, Altrecht, Netherlands; ^51^Faculty of Kinesiology, University of Calgary, Calgary, AB, Canada; ^52^Florida Institute for Human and Machine Cognition, Florida, IL, United States; ^53^United States Army War College, Carlisle, PA, United States; ^54^Decision Sciences Division, Defense Science and Technology Group, Adelaide, SA, Australia

**Keywords:** high performance, cognition, expert consensus, assessment, transdisciplinary

## Abstract

**Introduction:**

The ability to perform optimally under pressure is critical across many occupations, including the military, first responders, and competitive sport. Despite recognition that such performance depends on a range of cognitive factors, how common these factors are across performance domains remains unclear. The current study sought to integrate existing knowledge in the performance field in the form of a transdisciplinary expert consensus on the cognitive mechanisms that underlie performance under pressure.

**Methods:**

International experts were recruited from four performance domains [(i) Defense; (ii) Competitive Sport; (iii) Civilian High-stakes; and (iv) Performance Neuroscience]. Experts rated constructs from the Research Domain Criteria (RDoC) framework (and several expert-suggested constructs) across successive rounds, until all constructs reached consensus for inclusion or were eliminated. Finally, included constructs were ranked for their relative importance.

**Results:**

Sixty-eight experts completed the first Delphi round, with 94% of experts retained by the end of the Delphi process. The following 10 constructs reached consensus across all four panels (in order of overall ranking): (1) Attention; (2) Cognitive Control—Performance Monitoring; (3) Arousal and Regulatory Systems—Arousal; (4) Cognitive Control—Goal Selection, Updating, Representation, and Maintenance; (5) Cognitive Control—Response Selection and Inhibition/Suppression; (6) Working memory—Flexible Updating; (7) Working memory—Active Maintenance; (8) Perception and Understanding of Self—Self-knowledge; (9) Working memory—Interference Control, and (10) Expert-suggested—Shifting.

**Discussion:**

Our results identify a set of transdisciplinary neuroscience-informed constructs, validated through expert consensus. This expert consensus is critical to standardizing cognitive assessment and informing mechanism-targeted interventions in the broader field of human performance optimization.

## Background

A range of cognitive factors are considered key to attaining and sustaining optimal performance under pressure across application domains, such as the military, first responders, and competitive sport (Grier, 2012; Williams and Jackson, 2019; Aidman, 2020; Crameri et al., 2021). The terms used to define this field have remained relatively broad, such as High Performance Cognition introduced as an overarching construct for studies of human performance and skill acquisition (Cowley et al., 2020) covering a full range of conditions and skill levels, from novices to experts. As such they have not focused on the high-pressure^1^ element inherent across most performance domains. As the cognitive factors that underlie performance under pressure are distinct from those required within low-pressure contexts (e.g., Eysenck and Wilson, 2016), we extend the definition of high performance cognition to emphasize such high-pressure cognitive factors. That is, we will use a narrower definition of high performance cognition as cognitive factors that underpin performance under pressure. As an example of a candidate high performance cognitive factor, the ability to ignore task-irrelevant stimuli (distractors) is a key to staying focused on the task at hand under high-pressure conditions, which are known to challenge attentional processes (e.g., Janelle, 2002; Eysenck and Wilson, 2016; Martins, 2016). Despite high performance cognition being relevant across performance domains, to date, research in this space has progressed largely in domain-specific siloes. As such, it is not known how common these cognitive factors are across performance domains, nor can this question be answered easily given that domains tend to define and study these cognitive factors differently.

The emerging field of high performance cognition is in need of a coherent, unified framework to integrate existing knowledge and guide future research and progress (Cowley et al., 2020). There are a number of key benefits to having a unified framework high performance cognition. First, a unified framework can significantly enhance the efficiency of research progress through the field being able to benefit from learnings made across different domains (including avoiding repetition of mistakes; Fiore and Salas, 2008). Second, through the integration of knowledge across domains, a unified framework can enable a more comprehensive understanding of cognition in optimal performance *via* access to a wider range of operational contexts and populations. Critically, a limited context or scope of application can mask the influence of key moderators, resulting in misinterpretations (Burwitz et al., 1994). Third, a unified framework across performance domains will facilitate access to a wider range of resources and technologies to strengthen the field’s capacity to measure and optimize performance under pressure (e.g., see Williams et al., 2008 for a review). Finally, through integrating approaches and methods from different disciplines, a unified framework can facilitate new discoveries that are transformative, enabling significant leaps in thinking and new applications that transcend domain-specific boundaries (Fiore et al., 2008).

A barrier to establishing a unified framework of high performance cognition is the domain-specific nature of terminology and methods. Domain-specific terminology and methods make it difficult to integrate knowledge across domains, largely owing to the inability to compare findings that have been obtained through different methods. For instance, in sport, there has been extensive focus and progress achieved through domain-specific cognitive paradigms, such as those that gauge “anticipation,” i.e., the ability to predict what an opponent will do next (Williams and Jackson, 2019). Similarly, in the military, response inhibition and threat detection are commonly assessed in combat scenarios (e.g., the shoot/do not shoot paradigm; Biggs et al., 2021), while in aviation, situation awareness is typically measured using the domain-specific Situation Awareness Global Assessment Technique (Endsley, 2017). While domain-specific paradigms have strengths (e.g., Davids et al., 2015), the insights that they offer cannot be easily integrated across performance domains because the performance factors they assess confound the influence of domain-specific context (and experience within that context) with domain-general individual differences in high performance cognitive factors. To enable integration across different domains, the performance field is in need of a cognitive framework that uses comparable methods that are not confounded by domain-specific context or experience.

A framework that has the capacity to unify the current knowledge base through systematizing terminology and methods across performance domains is the Research Domain Criteria (RDoC; Insel et al., 2010). The RDoC emerged a framework to shift psychiatric research away from diagnostic and categorical understanding of psychiatric disorders and toward a more neuroscience-informed approach that conceptualizes psychopathology as reflecting dimensional, transdiagnostic neurobehavioral constructs. Supporting this shift toward transdiagnostic approaches, different diagnostic groups have been shown to share neurobiological underpinnings that correspond with functional dimensions independently of diagnostic label (for a review, see Cuthbert, 2022) In essence, diagnostic systems fundamentally misrepresent the mechanisms that drive psychopathology. In turn, research that studies diagnostic groups in a silo can produce misleading findings (owing to restricted range) as well as will hold back efforts to integrate knowledge across diagnoses to produce a more representative and accurate mechanistic understanding of psychopathology (Morris et al., 2022).

Arguably, the lessons from a transdiagnostic approach to the mechanisms that drive risk for psychopathology can be applied to develop a better understanding of the drivers of high performance. Just like a transdiagnostic approach can offer a more representative mechanistic understanding of psychopathology risk, a transdisciplinary approach can offer a more representative mechanistic understanding of high performance, i.e., one that does not confound domain-specific experience nor is limited by domain-specific bounds. Critically, understanding the neurocognitive mechanisms that drive high performance independently of domain will not only inform the detection of high performance potential in individuals but also guide the development of mechanism-targeted interventions to optimize performance across diverse operational settings (Fogarty et al., in press).

In addition to offering systematic terminology and measures to facilitate the integration of knowledge across different performance domains, the suitability of the RDoC as a framework for high performance cognition is highlighted by research showing that its constructs and measures are indeed relevant to high performance. Specifically, the RDoC lists 48 constructs and subconstructs that are grouped into six higher-order domains: Negative Valence Systems, Positive Valence Systems, Cognitive Systems, Systems for Social Processes, Arousal/Regulatory Systems, and Sensorimotor Systems (See Table 1 for more details). Whereas these constructs have to date been applied to understanding the mechanisms of risk and psychopathology, their dimensional range encompasses normal functioning and thereby may be implicated as driving potential for high performance in healthy individuals. Indeed, a number of RDoC constructs have already been linked to high performance. For instance, high performance has been linked to *Cognitive Control—Response Inhibition/Suppression* has been linked to high performance in sport (Vestberg et al., 2012; Chen et al., 2019) and military domains (Biggs and Pettijohn, 2022). Likewise, *Working Memory* and *Attention* have been linked to high performance in sport (Voss et al., 2010; Vestberg et al., 2017) and aviation (Causse et al., 2011; Gray et al., 2016). While research using RDoC-listed measures is relatively scarce compared to research using cognitive tasks that are not recommended by the RDoC (e.g., Kalén et al., 2021) or domain-specific paradigms such as those described previously, such research nonetheless highlights the relevance of the RDoC to high performance. In summary, the RDoC offers a system through which to study a wide range of cognitive processes that underlie variance in human functioning. It offers specific definitions of cognitive factors coupled with extensively-validated, neuroscience-informed measures that are not confounded by domain-specific context or experience, and which have been linked to high performance across different performance domains. These qualities make the RDoC an ideal system to bring together current knowledge from different performance domains and toward an integrated, unified framework of high performance cognition.

**Table 1:** RDOC constructs (see foot note 2).

Negative valence domain	Positive valence domain	Cognitive systems domain	Systems for social processes domain	Arousal/regulatory systems domain	Sensorimotor systems domain
Acute threat	Reward Responsiveness (*Reward Anticipation*; *Initial Response to Reward*; *Reward Satiation*)	Attention	Affiliation & Attachment	Arousal	Motor actions (*Action Planning & Selection*; *Sensorimotor Dynamics*; *Initiation*; *Execution*; *Inhibition & Termination*)
Potential threat	Reward Learning (*Probabilistic & Reinforcement Learning*; *Reward Prediction Error*; *Habit*)	Perception (*Visual Perception*; *Auditory Perception*; *Olfactory/Somatosensory/Multimodal Perception*)	Social Communication (*Reception of Facial Communication*; *Production of Facial Communication*; *Reception of Non-Facial Communication*; *Production of Non-Facial Communication*)	Circadian Rhythms	Agency and ownership
Sustained threat	Reward valuation (*Reward-Probability*; *Delay*; *Effort*)	Declarative memory	Perception & Understanding of self (*Agency*; *Self-Knowledge*)	Sleep and wakefulness	Habit
Loss		Language	Perception & Understanding of others (*Animacy Perception*; *Action Perception*; *Understanding Mental States*)		Innate motor patterns
Frustrative nonreward		Cognitive control (*Goal Selection, Updating, Representation, and Maintenance*; *Response Selection, Inhibition/Suppression*; *Performance Monitoring*)			
		Working memory (*Active Maintenance*; *Flexible Updating*; *Limited Capacity*; *Interference Control*)			

The current study used an RDoC-guided Delphi process to translate the diversity of expert knowledge across performance domains into a neuroscience-informed expert consensus. Specifically, the current Delphi sought to establish consensus across performance domains on the key cognitive factors that drive optimal performance in high-pressure operational contexts. The Delphi technique is a data-driven approach that implements rigorous and robust procedures to reach consensus among experts (Brown, 1968). Transdisciplinary consensus is necessary for building an integrated framework of high performance cognition to guide more coherent, far-reaching future progress across the performance field. A unified framework of high performance cognition supported by neuroscience evidence and uniformly-defined transdisciplinary constructs will also facilitate a broad agreement on the measurement tools for cognitive assessment as well as stimulating the development of neurocognitive mechanism-targeted interventions for performance optimization across diverse operational settings.

## Materials and methods

The current study employed RDoC-guided Delphi surveys to establish an expert consensus (Brown, 1968), on the key drivers of optimal performance under pressure. The Delphi method involves multiple iterations of an anonymous opinion survey, with each iteration incorporating participant feedback from the previous round. This process is repeated until a pre-determined level of consensus is reached (detailed below). Specifically, the current Delphi was an international, transdisciplinary, multi-panel Delphi study, with four panels representing experts from one of four performance domains: Military occupations (*Defense domain*); Sport and competition (*Competitive Sport domain*); First responder and other safety-critical, civilian high-stakes roles (*Civilian High-stakes domain*); and academics in areas directly relevant to understanding cognitive-affective processes that drive optimal performance under stress in dynamic, complex environments (*Performance Neuroscience*). Thus, there were three applied domain panels and one academic domain panel.

**Figure 1:**
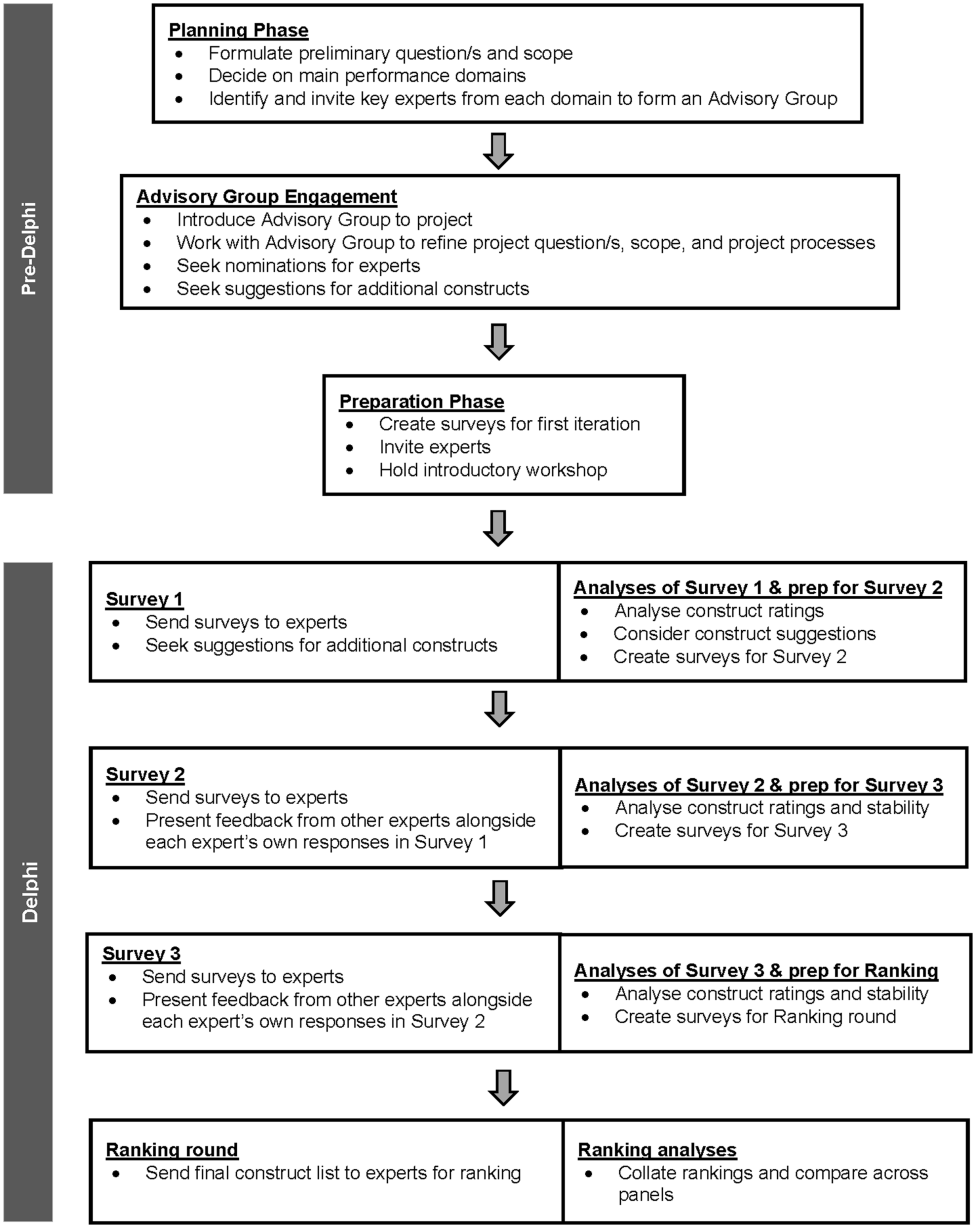
A visual representation of pre-Delphi and Delphi processes.

A pre-Delphi stage preceded the main Delphi data collection. The pre-Delphi stage included forming a Delphi Advisory group (*n* = 8) to guide our Delphi processes to ensure suitability of content and scope across all four domains. This study, including Advisory group participation in the pre-Delphi processes, was approved by the Monash University Ethics Committee and registered with Defence Science and Technology Group’s Low Risk Ethics Panel (DSTG LREP). All participants consented to participate. Pre-Delphi and Delphi sequence of events are summarized in Figure 1.

### Participants

Experts were identified through searches of key publications and organization websites as well as through suggestions made by experts. We aimed to recruit both practitioner and academic experts (as suggested by Baker et al., 2006). Criteria for inclusion as an expert practitioner included (a) having national or international recognition (e.g., coach for a national sport team) or (b) being suggested by at least two experts. Criteria for inclusion as an academic expert included (a) having at least three first- or senior-author peer-reviewed publications relevant to study *or* (b) being practitioner-researchers with at least one key publication *and* suggested by at least two experts. The list of experts was screened by the Advisory group members, who then made recommendations according to priority (based on study aims). We invited up to 20 experts per panel, which allowed for non-acceptance of invite and up to 50% drop-out without resulting in less than the required minimum of 10 per panel (Okoli and Pawlowski, 2004).

Invited experts who expressed interest in taking part were sent further information about the study by email, given a link to provide consent, and invited to attend an online Webinar-style information session led by the research team (which was recorded and made available for those who could not attend). This onboarding session described the background and rationale for the study, Delphi methodology, and an overview of the survey processes and instructions for completing the surveys. The recording was again sent to all participants prior to completing the first survey.

### Constructs

In addition to the 48 published RDoC constructs and subconstructs,^2^ additional constructs were suggested by expert participants, either during the Pre-Delphi phase (by Advisory group) or in Survey 1. An expert-suggested construct was included for consideration only if it met the following pre-determined criteria: (1) it was not a higher-order construct; (2) it was not adequately covered by existing RDoC constructs; and (3) there was evidence supporting an association between individual variations in performance on measures reflecting that construct and optimal performance under pressure. Constructs that failed to meet the above criteria were excluded from further consideration (See Figure S1; Supplementary materials). As the decision to include an expert-suggested construct depended on consideration of current research (to confirm it met the above criteria), when the team needed extra time to make a decision, the suggested construct was included for rating so as to not delay the survey schedule and excluded later.

### Procedure

Delphi surveys were distributed *via* personalized links and completed using Qualtrics and data analyses were conducted using SPSS ver. 27.

The key question presented to the experts throughout the Delphi surveys was: “How important do you think (RDoC/expert-suggested construct, e.g., attention) is to *optimal performance* in *dynamic* and *high-pressure* environments?” This question and corresponding *key term* definitions/features were decided through discussion with the Advisory group experts. The decision to use expert-guided definitions instead of using pre-existing definitions depended on the latter differed across domains. As the Advisory group included experts across the relevant domains, seeking their input to create Advisory-guided definitions enabled us to capture the defining features of key terms that applied across domains. These key terms and definitions were provided to all experts in the instructions as well as were accessible across the survey for all rounds. Specifically, *optimal performance* was defined according to three key features: (a) Implies sustained/consistent performance on multiple occasions under varying conditions; (b) May cover preparation, execution, and recovery phases; and (c) Applies to any level of technical expertise—from novices to experts. Further, when completing the Delphi surveys, experts were asked to imagine some typical scenarios that they considered representative of optimal performance in their field and to keep these in mind as they answered the questions (and using these same scenarios across survey iterations). *Dynamic environments* were defined according to two key features: (a) Have capacity to change; and (b) Are not static, consistent, or overly predictable. Finally, *high-pressure environments* were defined according to three key features: (a) Often involve high risk or capacity for significant loss or gain. In some contexts, this could be a life-or-death situation (could also be described as “high visibility,” “high expectation,” and “high demand”); (b) May include varying levels of complexity (involving uncertainty and ambiguity); and (c) May have multiple aspects requiring attention, tracking, decisions, and other cognitive manipulations. Ratings were given on a six-point Likert scale, which included the following response options: (1) Extremely important; (2) Very important; (3) Moderately important; (4) Slightly important; (5) Not important; and (6) Do not know/Unsure. The Delphi survey content (presented to experts in the first round) is included in the Supplementary materials.

We followed Delphi best practice guidelines for defining consensus and analyzing expert ratings and criteria (Trevelyan and Robinson, 2015). Specifically, consensus was determined as equal to or greater than 80% of experts voting a construct as important (i.e., extremely or very important; Putnam et al., 1995). Once a construct reached this level of consensus, it was removed from subsequent surveys and entered into the final construct list for that panel. Constructs rated as moderately, slightly, or *not* important by equal to or greater than 60% of experts were excluded from further consideration, as were any constructs whose rankings remained stable across rounds (assessed using Wilcoxon matched-pairs signed ranks tests; De Vet et al., 2005). Participants who responded “Do not know/Unsure” were not included in the stability analyses (for that construct). While there is very little research to inform the most suitable Likert scale response options to use in a Delphi (Drumm et al., 2022), we included a “Do not know/Unsure” option to avoid spurious changes in opinion over time.

Constructs not meeting these criteria were re-entered into the next survey round. This process was repeated until there were no constructs remaining, with all constructs having either reached consensus or been excluded Constructs were considered within panels, except for the constructs that were suggested at Round 1, which were entered into Round 2 across panels regardless of the panel that suggested them.

### Final ranking

At the conclusion of the survey rounds, experts were asked to rank the constructs that reached panel consensus against each other in their relative importance to optimal performance under pressure. This exercise created a priority list of constructs to guide an initial integrated framework of performance cognition.

## Results

Sixty-eight experts consented and completed the first Delphi round (Defense, *n* = 20; Competitive Sport, *n* = 18; Civilian High-Stakes, *n* = 16; and Performance Neuroscience, *n* = 14), and 64 experts stayed the whole 9-month long course of the study (retention rate = 94%). Thirty-four percent of experts were women. Experts’ primary affiliations spanned across 11 countries. Overall, the most common country of primary affiliation was Australia (44%), followed by the United States (28%) and the United Kingdom (10%). Table 2 presents gender, affiliation country, and retention rates by performance panel.

**Table 2:** Characteristics across the panels.

	Performance neuroscience	Defence	Civilian high-stakes	Competitive sport
Gender (Women, %)	36%	45%	19%	33%
Countries	Australia (57%), United States (21%), Germany*, Lebanon*, and Netherlands*	Australia (35%), United States (55%), and United Kingdom (10%)	Australia (44%), Canada (12.5%), Netherlands*, Norway*, United Kingdom (13%), and United States (19%)	Australia (44%), United Kingdom (17%), United States (11%), Belgium*, Canada*, Germany*, Ireland*, and Italy*
Retention	100%	95%	81%	100%

*denotes <10%.

**Figure 2:**
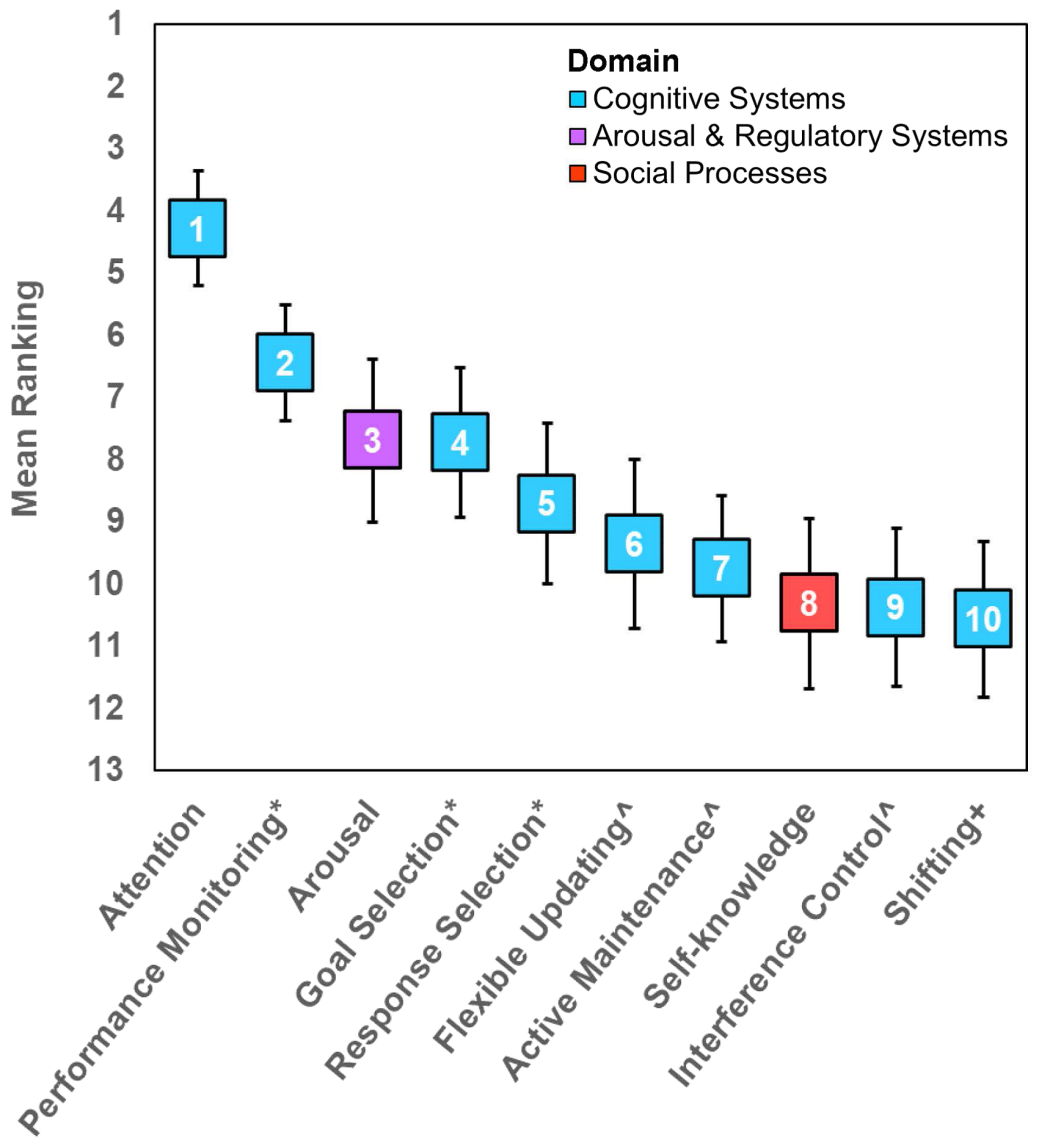
Mean ranking of the ten transdisciplinary constructs. Rank order is displayed within the corresponding marker. Error bars represent 95% Confidence Intervals. N.B. * denotes Cognitive Control subconstructs. ^ denotes Working Memory subconstructs. + denotes that Shifting was an expert-suggested construct (considered to belong in the Cognitive Systems Domain). ‘Goal Selection’ = Goal Selection; Updating; Representation; Maintenance. ‘Response Selection’ = Response Selection; Inhibition/Suppression.

Table 3 presents the panels’ ratings for all constructs at each survey round. Three rounds of surveys were required to reach the completion of the consensus process. The following 10 constructs reached consensus across all four panels (in order of overall ranking): (1) Attention; (2) Cognitive Control—Performance Monitoring; (3) Arousal and Regulatory Systems—Arousal; (4) Cognitive Control—Goal Selection, Updating, Representation, and Maintenance; (5) Cognitive Control—Response Selection and Inhibition/Suppression; (6) Working memory—Flexible Updating; (7) Working memory—Active Maintenance; (8) Perception and Understanding of Self—Self-knowledge; (9) Working memory—Interference Control, and (10) Expert-suggested—Shifting. Figure 2 presents the mean overall rankings of these 10 constructs. Table 4 presents all constructs that reached consensus, and their rankings per panel.

**Table 3:** All constructs, respective votes at each round, and outcomes.

Constructs		Performance domain	1	2	3
**RDoC DOMAIN: Negative Valence**					
Acute threat (Fear)		Perf. Neuroscience	64.3	71.4 ~	-
		Defence	45.0	63.2 ~	-
		Civilian High-stakes	43.8	78.6	56.3 ~
		Comp. Sport	44.4	72.2 ~	-
Potential threat (Anxiety)		Perf. Neuroscience	64.3	57.1 ~	-
		Defence	65.0	73.7 ~	-
		Civilian High-stakes	62.5	71.4 ~	-
		Comp. Sport	50.0	72.2 ~	-
Sustained threat		Perf. Neuroscience	50.0	50.0 ~	-
		Defence	35.0 #	-	-
		Civilian High-stakes	37.5 #	-	-
		Comp. Sport	72.2	66.7 ~	-
Loss		Perf. Neuroscience	35.7 #	-	-
		Defence	25.0 #	-	-
		Civilian High-stakes	12.5 #	-	-
		Comp. Sport	44.4	38.9 #	-
Frustrative nonreward		Perf. Neuroscience	21.4 #	-	-
		Defence	15.0 #	-	-
		Civilian High-stakes	25.0 #	-	-
		Comp. Sport	27.8 #	-	-
**RDoC DOMAIN: Positive Valence**					
Reward responsiveness	*Reward Anticipation*	Perf. Neuroscience	71.4	71.5 ~	-
		Defence	30.0 #	-	-
		Civilian High-stakes	18.8 #	-	-
		Comp. Sport	33.3 #	-	-
	*Initial Response to Reward*	Perf. Neuroscience	21.4 #	-	-
		Defence	5.0 #	-	-
		Civilian High-stakes	12.5 #	-	-
		Comp. Sport	11.1 #	-	-
	*Reward Satiation*	Perf. Neuroscience	35.7	35.7 #	-
		Defence	5.0 #	-	-
		Civilian High-stakes	12.5 #	-	-
		Comp. Sport	22.2 #	-	-
Reward learning	*Probabilistic & Reinforcement Learning*	Perf. Neuroscience	64.3	50.0 ~	-
		Defence	30.0 #	-	-
		Civilian High-stakes	37.5	50.0 ~	-
		Comp. Sport	38.9	50.0 ~	-
	*Reward Prediction Error*	Perf. Neuroscience	64.3	64.3 ~	-
		Defence	5.0 #	-	-
		Civilian High-stakes	25.0	14.3 #	-
		Comp. Sport	16.7 #	-	-
	*Habit*	Perf. Neuroscience	57.1	57.1 ~	-
		Defence	45.0	42.1 ~	-
		Civilian High-stakes	62.5	78.6 ~	-
		**Comp. Sport**	72.2	**83.3**	-
Reward valuation	*Reward (Probability)*	Perf. Neuroscience	50.0	35.7 ~	-	
		Defence	25.0 #	-	-	
		Civilian High-stakes	31.3	28.5 #	-	
		Comp. Sport	22.2 #	-	-	
	*Delay*	Perf. Neuroscience	21.4	21.4 #	-	
		Defence	5.0 #	-	-	
		Civilian High-stakes	18.8 #	-	-	
		Comp. Sport	22.2	22.2 #	-	
	*Effort*	Perf. Neuroscience	64.3	78.5 ~	-	
		Defence	45.0	57.9 ~	-	
		Civilian High-stakes	68.8	78.6 ~	-	
		**Comp. Sport**	66.7	**94.4**	-
**RDoC DOMAIN: Cognitive Systems**					
Attention		**Perf. Neuroscience**	**100.0**	-	-
		**Defence**	**100.0**	-	-
		**Civilian High-stakes**	**93.8**	-	-
		**Comp. Sport**	**100.0**	-	-
Perception	*Visual Perception*	Perf. Neuroscience	64.3	42.9 ~	-
		**Defence**	**90**	-	-
		**Civilian High-stakes**	**93.8**	-	-
		**Comp. Sport**	**100**	-	-
	*Auditory Perception*	Perf. Neuroscience	64.3	42.9	35.7 #
		Defence	60.0	68.5 ~	-
		**Civilian High-stakes**	**81.3**	-	-
		Comp. Sport	66.7	55.6	33.3 #
	*Olfactory/Somatosensory/Multimodal Perception*	Perf. Neuroscience	42.9	35.7 #	-
		Defence	35.0	21.1 #	-
		Civilian High-stakes	37.5 #	-	-
		Comp. Sport	38.9	27.8 #	-
Declarative memory		Perf. Neuroscience	71.4	71.4 ~	-
		**Defence**	75.0	**84.2**	-
		Civilian High-stakes	68.8	71.4 ~	-
		Comp. Sport	72.2	66.7 ~	-
Language		Perf. Neuroscience	71.4	64.3 ~	-
		**Defence**	75.0	**89.5**	-
		Civilian High-stakes	68.8	78.6 ~	-
		Comp. Sport	38.9	38.9 ~	-
Cognitive control	*Goal Selection*; *Updating, Representation, & Maintenance*	**Perf. Neuroscience**	**100.0**	-	-
		**Defence**	**95.0**	-	-
		**Civilian High-stakes**	**87.5**	-	-
		**Comp. Sport**	**83.3**	-	-
	*Response Selection*; *Inhibition/Suppression*	**Perf. Neuroscience**	**92.9**	-	-
		**Defence**	**95.0**	-	-
		**Civilian High-stakes**	**87.5**	-	-
		**Comp. Sport**	**83.3**	-	-
	*Performance Monitoring*	**Perf. Neuroscience**	*	**92.9**	-
		**Defence**	*	**94.7**	-
		**Civilian High-stakes**	*	**100.0**	-
		**Comp. Sport**	*	**94.4**	-
Working memory	*Active Maintenance*	**Perf. Neuroscience**	**85.7**	-	-	
		**Defence**	75.0	**89.5**	-	
		**Civilian High-stakes**	**81.3**	-	-	
		**Comp. Sport**	77.8	**88.9**	-	
	*Flexible updating*	**Perf. Neuroscience**	**100.0**	-	-	
		**Defence**	**95.0**	-	-	
		**Civilian High-stakes**	**81.3**	-	-	
		**Comp. Sport**	**88.9**	-	-	
	*Limited Capacity*	Perf. Neuroscience	57.1	71.4 ~	-	
		Defence	50.0	68.4 ~	-	
		Civilian High-stakes	56.3	50.0 ~	-	
		Comp. Sport	33.3	72.2 ~	-	
	*Interference Control*	**Perf. Neuroscience**	**92.9**	-	-	
		**Defence**	**85.0**	-	-	
		**Civilian High-stakes**	**81.3**	-	-
		**Comp. Sport**	**88.9**	-	-
**RDoC DOMAIN: Systems for Social Processes**					
Affiliation & attachment		Perf. Neuroscience	35.7 #	-	-
		Defence	70.0	78.9 ~	-
		Civilian High-stakes	43.8	50.0 ~	-
		Comp. Sport	33.3 #	-	-
Social Communication	*Reception of Facial Communication*	Perf. Neuroscience	57.1	64.3 ~	-
		Defence	40.0 #	-	-
		Civilian High-stakes	68.8	78.6 ~	-
		Comp. Sport	38.9	22.2 #	-
	*Production of Facial Communication*	Perf. Neuroscience	42.9	21.4 #	-
		Defence	35.0 #	-	-
		Civilian High-stakes	37.5 #	-	-
		Comp. Sport	11.1 #	-	-
	*Reception of Non-Facial Communication*	Perf. Neuroscience	42.9	35.7 #	-
		Defence	45.0	63.2 ~	-
		Civilian High-stakes	56.3	64.3 ~	-
		Comp. Sport	22.2 #	-	-
	*Production of Non-Facial Communication*	Perf. Neuroscience	28.6 #	-	-
		Defence	40.0 #	-	-
		Civilian High-stakes	31.3 #	-	-
		Comp. Sport	16.7 #	-	-
Perception & understanding of self	*Agency*	Perf. Neuroscience	64.3	71.4 ~	-
		Defence	55.0	73.7 ~	-
		Civilian High-stakes	43.8	64.3 ~	-
		Comp. Sport	77.8	66.7 ~	-
	*Self-Knowledge*	**Perf. Neuroscience**	**92.9**	-	-
		**Defence**	**85.0**	-	-
		**Civilian High-stakes**	68.8	**100.0**	-
		**Comp. Sport**	**88.9**	-	-
Perception & understanding of others	*Animacy Perception*	Perf. Neuroscience	42.9	50.0 ~	-	
		Defence	35.0 #	-	-	
		Civilian High-stakes	37.5	21.4 #	-	
		Comp. Sport	38.9	22.2 #	-	
	*Action Perception*	Perf. Neuroscience	71.4	78.6 ~	-	
		Defence	55.0	78.9 ~	-	
		Civilian High-stakes	56.3	78.6 ~	-	
		**Comp. Sport**	77.8	**83.3**	-	
	*Understanding Mental States*	Perf. Neuroscience	78.6	78.6	-	
		**Defence**	65.0	**89.5**	-	
		**Civilian High-stakes**	**87.5**	-	-	
		**Comp. Sport**	72.2	**88.9**	-
**RDoC DOMAIN: Arousal/Regulatory Systems**					
Arousal		**Perf. Neuroscience**	**92.9**	-	-
		**Defence**	**80.0**	-	-
		**Civilian High-stakes**	75.0	**92.9**	-
		**Comp. Sport**	**83.3**	-	-
Circadian rhythms		Perf. Neuroscience	57.1	57.1 ~	-
		Defence	50.0	47.4 ~	-
		Civilian High-stakes	50.0	50.0 ~	-
		Comp. Sport	44.4	50.0 ~	-
Sleep and wakefulness		Perf. Neuroscience	71.4	50.0 ~	-
		Defence	70.0	73.7 ~	-
		Civilian High-stakes	62.5	64.3 ~	-
		Comp. Sport	66.7	61.1 ~	-
**RDoC DOMAIN: Sensorimotor Systems**					
Motor actions	*Action Planning & Selection*	**Perf. Neuroscience**	71.4	**85.7**	-
		Defence	65.0	73.7 ~	-
		Civilian High-stakes	56.3	78.6 ~	-
		**Comp. Sport**	**94.4**	-	-
	*Sensorimotor Dynamics*	Perf. Neuroscience	42.9	42.9 ~	-
		Defence	40.0	21.1 #	-
		Civilian High-stakes	37.5	14.3 #	-
		**Comp. Sport**	**83.3**	-	-
	*Initiation*	Perf. Neuroscience	57.1	50.0 ~	-
		Defence	30.0 #	-	-
		Civilian High-stakes	37.5	42.9 ~	-
		Comp. Sport	77.8	77.8 ~	-
	*Execution*	Perf. Neuroscience	64.3	64.3 ~	-
		Defence	50.0	52.6 ~	-
		Civilian High-stakes	62.5	78.6 ~	-
		**Comp. Sport**	**94.4**	-	-
	*Inhibition & Termination*	Perf. Neuroscience	64.3	78.6 ~	-
		Defence	55.0	63.2 ~	-
		Civilian High-stakes	50.0	64.3 ~	-
		Comp. Sport	61.1	72.2 ~	-
Agency and ownership		Perf. Neuroscience	42.9	50.0 ~	-
		Defence	35.0 #	-	-
		Civilian High-stakes	31.3 #	-	-
		Comp. Sport	77.8	61.1 ~	-
Habit		Perf. Neuroscience	42.9	42.9 ~	-
		Defence	50.0	52.6 ~	-
		Civilian High-stakes	56.3	71.4 ~	-
		**Comp. Sport**	**88.9**	-	-
Innate motor patterns		Perf. Neuroscience	7.1 #	-	-	
		Defence	15.0 #	-	-	
		Civilian High-stakes	31.3	35.7 #	-	
		Comp. Sport	22.2	22.2 #	-
**Expert-suggested constructs**					
Processing speed		Perf. Neuroscience	71.4	78.6 ~	-
		**Defence**	**90.0**	-	-
		**Civilian High-stakes**	**87.5**	-	-
		**Comp. Sport**	**88.9**	-	-
Shifting		**Perf. Neuroscience**	78.6	**85.7**	-
		**Defence**	**95.0**	-	-
		**Civilian High-stakes**	75.0	**92.9**	-
		**Comp. Sport**	**83.3**	-	-
Interoception		Perf. Neuroscience	-	57.1	57.1 ~
		Defence	-	63.2	45.0 ~
		Civilian High-stakes	-	28.6	38.5 #
		**Comp. Sport**	-	72.2	**83.4**
**Later excluded**					
Discomfort tolerance		**Perf. Neuroscience**	**85.7**	-	-
		**Defence**	**100.0**	-	-
		**Civilian High-stakes**	**87.5**	-	-
		**Comp. Sport**	77.8	**88.9**	-
Mental fatigue		**Perf. Neuroscience**	-	**85.7**	-
		**Defence**	-	73.7	**80.0**
		**Civilian High-stakes**	-	**85.7**	-
		**Comp. Sport**	-	**88.9**	-
Cognitive motor interference		Perf. Neuroscience	-	42.9	35.7 #
		Defence	-	10.5 #	-
		Civilian High-stakes	-	28.6	18.8 #
		Comp. Sport	-	72.2	61.1 ~
Procedural memory		Perf. Neuroscience	-	64.3	78.6 ~
		Defence	-	57.9	65.0 ~
		Civilian High-stakes	-	50.0	62.6 ~
		Comp. Sport	-	66.7	61.1 ~

Bolded font indicates consensus was reached. “~” denotes stability was reached. “#” denotes exclusion based on low importance.

**Table 4:** Construct rankings across panels.

Domain—Construct−*Subconstruct*	Perf. neuroscience	Defence	Civilian high-stakes	Comp. sport
CS—Attention	3	1	1	1
CS—Cognitive Control – *Performance Monitoring*	5	4	2	2
A/RS—Arousal	11	2	4	6
CS—Cognitive Control – *Goal Selection*; *Updating, Representation, & Maintenance*	1	6	5	7
CS—Cognitive Control – *Response Selection*; *Inhibition/Suppression*	2	5	6	14
CS—Working Memory – *Flexible Updating*	4	7	3	19
CS—Working Memory – *Active Maintenance*	8	12	9	11
SfSP—Perception and Understanding of Self - *Self-knowledge*	9	11	13	8
CS—Working Memory – *Interference Control*	6	13	8	15
ES—Shifting	10	8	11	13
ES—Processing Speed	–	3	7	10
CS—Perception – *Visual Perception*	–	9	10	4
SfSP—Perception and Understanding of Others – *Understanding Mental States*	–	10	12	16
SS—Motor Actions – *Action Planning and Selection*	7	–	–	5
CS—Language	–	14	–	–
CS—Declarative Memory	–	15	–	–
SS—Motor Actions – *Execution*	–	–	–	3
PVS—Reward Valuation – *Effort*	–	–	–	9
SfSP—Perception and Understanding of Others – *Action Perception*	–	–	–	12
ES—Interoception	–	–	–	17
SS—Motor Actions – *Sensorimotor Dynamics*	–	–	–	18
PVS—Reward Learning – *Habit*	–	–	–	20
SS—Habit	–	–	–	21
CS—Perception—*Auditory Perception*	–	–	14	-

Three constructs reached consensus across all three applied domains, including (1) Processing Speed (expert suggested) and (2) Visual Perception (from Cognitive Systems), and Perception and Understanding of Others—Understanding Mental States (from Systems for Social Processes). The Defense panel uniquely rated Language and Declarative Memory (from Cognitive Systems) as important. The Civilian High-Stakes panel uniquely rated Auditory Perception (from Cognitive Systems) as important. The Competitive sport panel uniquely rated the greatest number of constructs (i.e., 7), with their top-ranking unique construct being Motor Actions—Execution (from Sensorimotor Systems).

## Discussion

The aim of this study was to achieve a neuroscience-guided expert-based consensus on the cognitive constructs that are a key to optimal performance under pressure across multiple performance domains. This consensus is an important first step toward building the foundations for an integrated transdisciplinary framework of high performance cognition to guide coherence of future research and progress across the performance field. A transdisciplinary expert consensus was reached for 10 such constructs, as judged by academic and practice experts within all four Delphi panels. Seven of these transdisciplinary constructs were from the RDoC Cognitive Systems domain, with Attention being the top-voted transdisciplinary construct. Other RDoC constructs came from the Systems for Social Processes domain (i.e., self-knowledge) and the Arousal/Regulatory Systems domain (i.e., arousal). Shifting (of attentional or task set) was the only non-RDoC construct that reached transdisciplinary consensus.

The finding that attention ranked most important across domains is in line with the extensive focus dedicated to attention within each performance domain as well as its interaction with high-pressure contexts. For instance, in sport, there is a prominence of attentional models to explain performance under pressure (Nideffer, 1987; Eysenck and Wilson, 2016; Moran, 2016), such as the Attentional Control Theory: Sport (ACTS; Eysenck and Wilson, 2016), which was developed specifically to explain how attentional processes can be influenced by the high-pressure conditions that are inherent in sport, as well as other performance contexts. Attention is also a key process in situational awareness (Endsley, 1988), one of the most widely investigated cognitive constructs in aviation. Finally, attention is one of the most extensively studied outcomes in military cognitive enhancement research (Kelley et al., 2019). Critically, the fact that attention has been approached from such different perspectives across different domains highlights the potential of an integrated framework to enable such progress to be translated into a common language and applied to benefit other domains. For instance, an integrated, neuroscience-based framework could be applied to translating the ACTS model into a common language, thereby enabling its application across performance domains.

A finding that warrants special mention is that of self-knowledge being considered a key cognitive factor for optimal performance under pressure across all domains. While self-knowledge’s relevance to optimal performance under pressure may be assumed *via* its contribution to higher-order concepts such as emotion regulation (e.g., Barrett et al., 2001), it has very rarely been examined (in the performance field) using cognitive or otherwise objective methods. In fact, there are no studies in the performance field that have used the RDoC-listed paradigm for this construct (i.e., self-referential memory paradigm). The fact that experts across all performance domains agreed that self-knowledge is a key to optimal performance combined with the lack of neurocognitive research in this space presents an outstanding opportunity for future research to create new knowledge on and/or solutions harnessing self-knowledge that could change the landscape of the performance field.

As explained in the introduction, an advantage of using the RDoC to guide an expert consensus on key constructs of high performance cognition is the extensive neuroscientific evidence upon which it is based, including a range of validated measures to index level of functioning on corresponding constructs. For instance, RDoC suggests response inhibition can be measured *via* the Stop-Signal Task (among other select measures). Unfortunately, the majority of current measures listed by the RDoC for corresponding constructs have only been validated in relation to risk of, and/or current psychopathology. It is yet to be determined whether many of the RDoC-listed measures will be sensitive to individual differences among high-performing individuals at the upper end of the normative distribution (according to similarly rigorous measurement standards). This is a crucial next step in building a high performance cognition framework that will systematize cognitive assessment methods.

Another key step moving forward is to delineate the scope and content of certain RDoC constructs as they relate to high performance cognition, such as attention. Whereas attention can be considered a more basic process than, say, situational awareness, it is itself unlikely to be sufficiently precise to guide meaningful mechanistic insights. Indeed, the RDoC notes different attentional processes that fall within the attention construct, including selective and divided attention. Further, the RDoC differentiates between *sustained attention*, which is allocated to goal maintenance (a sub-construct of cognitive control), and *vigilance,* which they keep under attention (albeit this is noted informally, within RDoC Proceedings). While vigilance, selective attention, and divided attention are recognized (informally) as distinct attention-related processes by the RDoC (NIMH, 2011), they have not yet been formally listed as attention sub-constructs. Given the primary role of attention in performance, the performance field is ideally placed to lead the way toward delineating separable neural circuits for different types of attention.

A third priority for future research is to understand how the constructs highlighted through this Delphi study combine and interact to produce important higher order constructs, such as situational awareness and adaptability. Whereas the current Delphi study focused on basic cognitive processes of performance under pressure (as opposed to higher-order constructs such as situational awareness), this was not intended to detract from the importance of higher-order constructs. In fact, a main rationale behind the need to better understand the key basic processes that drive performance under pressure is to enable a more precise future understanding of higher-order processes and their measurement. Similarly, understanding how these cognitive processes interact with high-pressure environments to support optimal performance is a key to informing interventions for optimization of cognitive resilience (Flood and Keegan, 2022). Understanding how specific cognitive processes interact with context and state factors will be critical for informing precise mechanism-targeted interventions. For instance, understanding and measuring situational awareness in a way that reflects the different contributions of specific/basic cognitive factors (e.g., attention, working memory) means that when assessed across different contexts (under time pressure, under threat, in sport, in aviation, etc.) or across different individuals, any differences (or lack of) in overall situational awareness can be understood more precisely. For instance, two individuals might show comparable overall situational awareness; however, the specific cognitive factors contributing to their overall situational awareness might differ considerably. Therefore, these individuals could respond very differently to training, depending on the focus of the training and the extent to which it matched their profile. In contrast, if their situational awareness abilities could be understood in terms of the combination of basic cognitive processes, then such knowledge could be used to develop personalized mechanism-targeted interventions such that precise cognitive processes can be selectively targeted. The same principle applies to situational awareness across different operational contexts. To this end, work is currently underway to create assessments of these cognitive interactions through integrated tasks wherein separate cognitive processes can be assessed in the context of other processes (controlled through task selection) while keeping their measurement separable (Wells et al., 2021; Kucina et al., 2022).

### Limitations

There is a lack of generally agreed upon standards of Delphi best practices for analyzing expert ratings and defining consensus criteria, which can leave many key decisions at the discretion of the researchers leading it (Mitchell, 1991; Fink-Hafner et al., 2019). We addressed this uncertainty through detailed and transparent reporting as well as being guided by the available (albeit limited) research on what constitutes good practice in Delphi methodology (Okoli and Pawlowski, 2004; Hussler et al., 2011; Trevelyan and Robinson, 2015). Another potential limitation of the current Delphi is that levels of familiarity with the RDoC varied across expert subpanels. This was addressed early on and throughout the project through sending onboarding materials and holding workshops to explain the background and RDoC concepts, and recapping all the key points and definitions at each survey round. Finally, limitations pertaining to the representativeness of the current expert sample should be considered. For instance, our panel was dominated by experts from Australia, United States, and Europe. While we did send invitations to a number of experts from Asian countries (e.g., Singapore), this did not result in uptake. Future studies examining the opinions of experts from non-European countries will be important to confirm the current findings or highlight cultural differences in expert options. Another feature of the current study that might be considered to limit the representativeness of our findings is the selection of our panels. While the panels were chosen with the aim of ensuring maximal coverage of occupational groups and expertise pertaining to performance under pressure, the civilian high-stakes roles panel included a diverse range of occupations, from first responders to medical and aviation experts, potentially with insufficient numbers of experts within these sub-domains. However, as domains could continue to be broken down into smaller sub-domains, we believe that the conceptual grouping we used was more meaningful for our purposes than opting for more narrow occupational groups. Once an integrated framework gets developed, future research can examine similarities and differences across these sub-domains.

Despite the limitations inherent to the Delphi technique, its use in the current study is arguably one of its major strengths. First, as explained at the outset, the Delphi method is a rigorous data-driven approach that implements robust procedures to reach expert consensus. Second, the Delphi technique was uniquely suitable to achieve our aim to develop a trans-disciplinary consensus—as distinct from reviewing the evidence across the performance domains in search of the key constructs of high performance cognition. The latter would have been limited by the diversity of methods and terminology across the different domains. Rather, our aim was to transform the diversity and breadth of knowledge that exists across performance domains (which have been separated by domain silos) into a set of transdisciplinary, neuroscience-informed constructs based on expert agreement. An RDoC-guided Delphi method was perfectly suited to meet this goal. Indeed, this method has been used to create transformative frameworks in other fields faced with similar challenges (Yücel et al., 2019, 2021).

## Conclusion

In conclusion, this Delphi study has produced a transdisciplinary expert consensus on the cognitive drivers of optimal performance under pressure across multiple performance domains. The resulting set of neuroscience-informed constructs, applicable within and across performance domains, can serve as an integrated framework of high performance cognition to facilitate shared progress in the broader field of human performance. An integrated framework of high performance cognition has potential to bolster a broad agreement on, and stimulate the development of (1) mechanism-sensitive measurement tools for precise cognitive assessment and (2) cognitive mechanism-targeted interventions to build cognitive fitness and optimize performance under high pressure. Finally, the current findings are of direct relevance to a broader understanding of optimal performance under pressure across operational environments as well as optimal functioning generally. That is, the ability to perform optimally under pressure of benefit to everyone, from an athlete competing in the Olympics to a parent dealing with a child’s asthma attack. Through establishing the foundations for an integrated framework of high performance cognition, the current findings can facilitate future progress that transcends disciplinary bounds and inform systematic approaches to measuring and improving individuals’ capacities to adapt to a wide range of challenges.

## Data availability statement

The raw data supporting the conclusions of this article will be made available by the authors, without undue reservation.

## Ethics statement

The studies involving human participants were reviewed and approved by Monash University Human Research Ethics Committee Defense Science Technology Group’s Low Risk Ethics Panel (DSTG LREP). The patients/participants provided their written informed consent to participate in this study.

## Author contributions

MY and EA conceived the study idea. LA and RK coordinated data collection and analyzed the data. LA wrote the first draft. All authors contributed to the Delphi and consensus processes, provided feedback on drafts, as well as read and approved the final manuscript.

## Funding

This study was funded by a research agreement MYIP:9522 from the Australian Department of Defence, under the Human Performance Research Network (HPRNet). RK and LA have received funding from the HPRNet and David Winston Turner Endowment Fund. MY received funding from Monash University, and Australian Government funding bodies: the National Health and Medical Research Council (NHMRC; including Fellowship #APP1117188), the Australian Research Council (ARC), the Australian Defence Science and Technology (DST) Group, and the Department of Industry, Innovation and Science (DIIS). He has also received philanthropic donations from the David Winston Turner Endowment Fund, Wilson Foundation. AH received funding from the Australian Army (Cognitive Testing Grant). JA has received funding from the Canadian Institutes for Health Research (CIHR) Team Grant [Mental Wellness in Public Safety - Police (433650)]. JG is supported by a NHMRC Project Grant (1122816). MC received funding from Science Foundation Ireland grant 13/RC/2094_P2 and co-funded under the European Regional Development Fund through the Southern & Eastern Regional Operational Programme to Lero - the Science Foundation Ireland Research Centre for Software (www.lero.ie). WK was funded by grants from the U.S. Department of Defense. PH received funding from the Federal Aviation Administration (FAA). SK received funding from the Australian Army HQ (RA G208313, 2020), and previously from the DST Group, and from the University of Sydney. AT received research funding from the Cooperative Research Centre for Alertness, Safety and Productivity, BHP, Rio Tinto, Shell and VicRoads. SD has received funding from US Department of Defense, DST Group, NHMRC; Member, Board of Advisors Eisai Australia Pty Ltd. SL has received funding from the ARC, Defence Science Centre, DST Group, and the Research Network for Undersea Decision Superiority. TV was funded by the DST Group and the Australian Army. The funding sources had no influence or involvement the design, management, data analysis, presentation, or interpretation and write-up of the data.

## Conflict of interest

VM was employed by Mindflex Group Ltd. JG is a stockholder in MAP Biotech Pty Ltd. SC has received speakers fees Janssen-Cilag Australia, Lundbeck Otsuka Australia, Servier Australia; Investigator Initiated research funding Janssen-Cilag Australia; Lundbeck Otsuka Australia; Advisory Boards Lundbeck Otsuka Australia. AT has received research funding from BHP, Rio Tinto, and Shell. SD is a Member of the Board of Advisors Eisai Australia Pty Ltd. MY has received payments in relation to court-, expert witness-, and/or expert review-reports. JD was employed by Florida Maxima Corporation.

The remaining authors declare that the research was conducted in the absence of any commercial or financial relationships that could be construed as a potential conflict of interest.

## Publisher’s note

All claims expressed in this article are solely those of the authors and do not necessarily represent those of their affiliated organizations, or those of the publisher, the editors and the reviewers. Any product that may be evaluated in this article, or claim that may be made by its manufacturer, is not guaranteed or endorsed by the publisher.

## Footnotes

^1^Generally, the term ‘high pressure’ is intended to cover a range of conditions, such as threat, ambiguity, change, and performance expectations, that characterize operational contexts across performance domains (Bartone et al., 1998; Nieuwenhuys and Oudejans, 2017).

^2^ https://www.nimh.nih.gov/research/research-funded-by-nimh/rdoc/constructs/
