# Erratum: Building a transdisciplinary expert consensus on the cognitive drivers of performance under pressure: an international multi-panel Delphi study

**DOI:** 10.3389/fpsyg.2023.1236133

**Published:** 2023-06-20

**Authors:** Frontiers Production Office

**Affiliations:** Frontiers Media SA, Lausanne, Switzerland

**Keywords:** high performance, cognition, expert consensus, assessment, transdisciplinary

Due to a production error, an author's name was incorrectly spelled as “Kristin J. Heatown.” The correct spelling is “Kristin J. Heaton.”

The publisher apologizes for this mistake. The original article has been updated.

