# Supplementary material for: Building a transdisciplinary expert consensus on the cognitive drivers of performance under pressure: An international multi-panel Delphi study
Source: Front Psychol. 2023 Jan 18;13:1017675. doi: 10.3389/fpsyg.2022.1017675 (PMC9901503; doi:10.3389/fpsyg.2022.1017675)
Supplement: Supplementary file 1 [file Image_1.pdf]

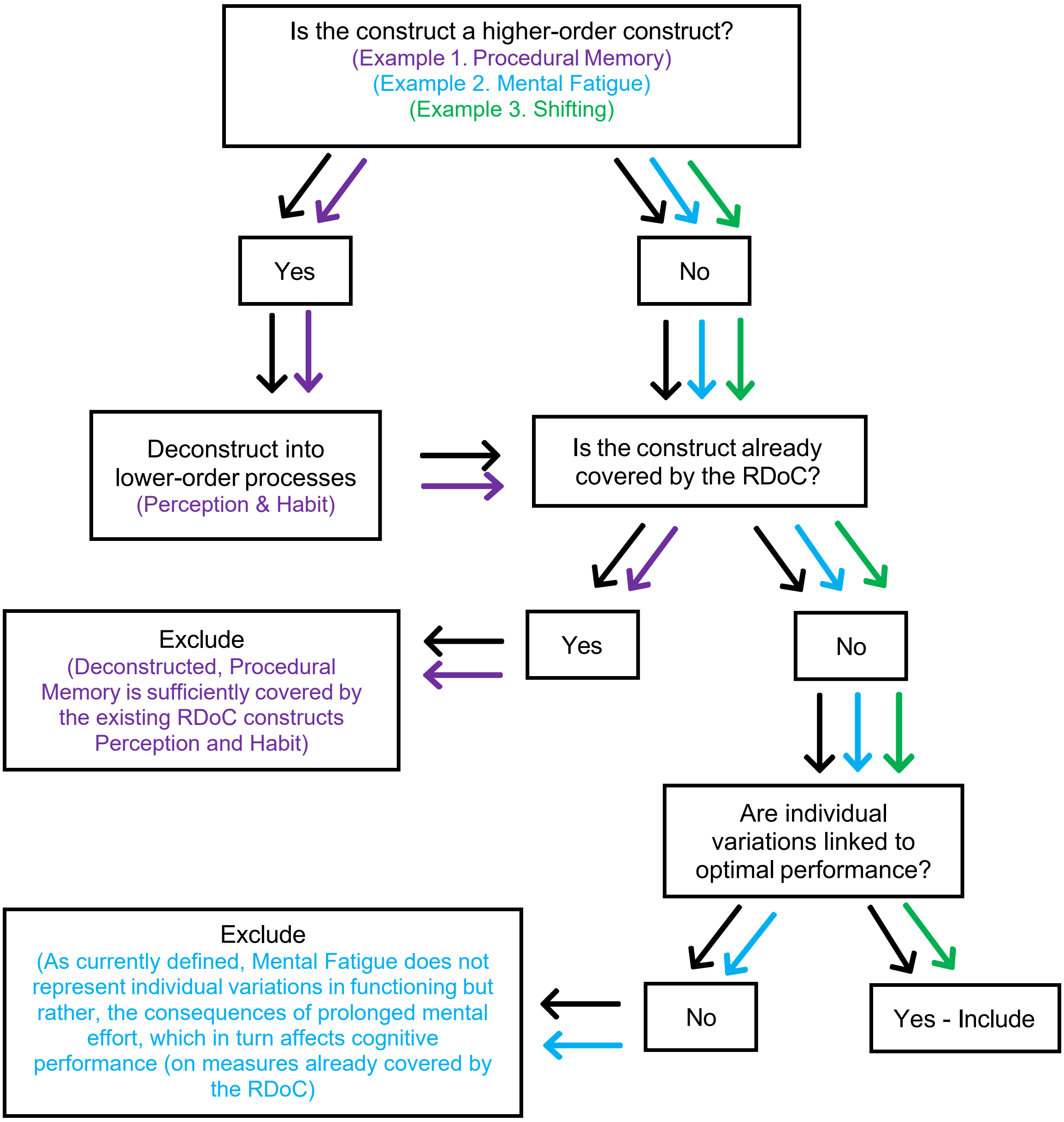

Figure S1. Decision making sequence for including expert-suggested constructs into the Delphi survey, including three examples of decisions made (represented by different colors).
