# Supplementary material for: Building a transdisciplinary expert consensus on the cognitive drivers of performance under pressure: An international multi-panel Delphi study
Source: Front Psychol. 2023 Jan 18;13:1017675. doi: 10.3389/fpsyg.2022.1017675 (PMC9901503; doi:10.3389/fpsyg.2022.1017675)
Supplement: Supplementary file 2 [file Presentation_1.PDF]

Instructions

Name (Title, First, Last)

In what field do you work? (If transdisciplinary, detail the fields with the primary first)

With which category do you best align?

- ◯ Defence
- ◯ Sport and Competition
- ◯ High-stakes roles (Including aviation, medical & first responder roles)
- ◯ Applied Cognitive Neuroscience (Including cognitive and affective mechanisms)

INTRODUCTION

Optimal performance in dynamic and high-pressure environments is considered critical in many occupations such as competitive sport, first responder, law enforcement and military professions. While it is broadly acknowledged that performance in these contexts depends on multiple aspects of cognitive functioning (collectively comprising cognitive fitness), their exact nature and relative importance remain unclear. Our project aims to develop an expert consensus on the key dimensions of cognitive fitness, broadly applicable to diverse "performance under pressure" contexts. This consensus will inform a more systematic approach to extending the assessment of cognitive functioning from deficit to high performance, as well as to developing targeted interventions to modify cognitive performance through treatment, training and augmentation.

Our research question is "What are the psychological constructs that underlie optimal performance in dynamic and high-pressure environments?"

We will therefore be asking you "How important is [given construct] to optimal performance in dynamic and high-pressure environments?" from the perspective of **your expert field** for each RDoC construct and expert suggested constructs.

DEFINITIONS

Constructs (A measurable something that can be measured with multiple metrics. For this project, a "psychological construct" represents a specified dimension of behaviour that can be measured through a range of methods, i.e. self-report, response patterns, biomarkers etc.)
- Influence individual differences in real-time performance execution
- Are the most fundamental level of the construct (i.e. the building block, not a higher-order construct)
- Can or could be measured

Optimal Performance
- Implies sustained / consistent performance on multiple occasions under varying conditions
- Can cover preparation, execution, and recovery phases
- Applies to any level of technical expertise – from novices to experts

Dynamic environments
- Has the capacity to change
- Is not static, consistent, or overly predictable

High-pressure environments
- Often involves high risk or capacity for significant loss or gain. In some contexts, this could be a life or death situation (could also be described as 'high visibility', 'high expectation', 'high demand')
- May include varying levels of complexity (involving uncertainty, ambiguity)
- May have multiple aspects requiring attention, tracking, decisions, and other cognitive manipulations

Before we begin...

Before we begin...

We recognise that there are many scenarios that require optimal performance in your field and that each scenario might elicit different construct ratings.

We therefore ask you to imagine some typical scenarios that you would consider representative of optimal performance in your field. Have about three scenarios jotted down or ready in the forefront of your mind when you do the survey. They don't have to be exclusive or exclusionary of other scenarios, but they may help you whilst completing the survey.

Once you have a few scenarios where you think you can pinpoint optimal performance, please click the next arrow.

Instructions Part 2

INSTRUCTIONS

RDoC CONSTRUCTS

We ask you to rate the constructs listed in the RDoC according to their importance to optimal performance in dynamic and high-pressure environments, in your field.

For each construct, you can provide your rationale for rating as you have. This may be particularly important if you feel strongly about the rating you have provided. These comments will be shared anonymously with the Delphi panel in the subsequent round and have the potential to sway others' ratings on the construct. Since such comments will be shared widely amongst transdisciplinary experts, please try to keep your language communicable to educated lay persons.

Please answer all questions to the best of your ability, or simply reply "Don't know / Unsure" where you do not feel you have sufficient knowledge.

In subsequent rounds you will have the opportunity to revise your answers in light of the group's ratings and comments.

Please click on the construct name to be taken to the RDoC website for further enquiry. The description and behaviour provided by RDoC is included in the question if available.
At the start of each new RDoC domain page, you will be able to open a pdf to the definitions of the key terms in the question.

At the end of this questionnaire you will have the opportunity to offer additional psychological constructs to your original suggestions, provide additional comments and to review your ratings.

Importance: Negative Valence Systems

DOMAIN: Negative Valence Systems

Desciption: Negative Valence Systems are primarily responsible for responses to aversive situations or context, such as fear, anxiety, and loss.

Constructs:

- Acute Threat "Fear"
- Potential Threat "Anxiety"
- Sustained Threat
- Loss
- Frustrative Nonreward

Definitions of key terms.pdf

Comments from round 1 showed that people generally considered the relevance of the negative valence constructs in one of two ways:

1. The ability to perform optimally despite negative valence factors such as fear and anxiety
2. The effect of negative valence factors themselves on performance (either enhancing/optimising or disrupting/degrading it).

From now on, can we ask you to **concentrate on option 2**.

As an example for acute threat ('fear'), consider two candidates going through Defence recruitment. As part of the selection process, they must complete a complex cognitive task in the presence of a threat cue.

Under neutral conditions (no threat cues) Candidate A performs the task to high standards. In the presence of a threat cue, the candidate demonstrates an **unusually strong fear response** and performs the task to **adequate standards**.

Candidate B also performs the task to high standards under neutral conditions (no threat cues). In the presence of a threat cue, they show a **normal fear response** and perform the cognitive task to the same **high standards**.

In these scenarios, one may say that acute threat ('fear') is relevant to optimal performance. Specifically, **all other things being equal**, Candidate A's elevated fear response interfered with their ability to perform the cognitive task to high standards.

When considering the relevance/importance of negative valence constructs, can we ask you to focus on their impact on performance while controlling for potential confounds (i.e., all other things being equal).

CONSTRUCT	Acute Threat "Fear"
Description (RDoC)	Activation of the brain's defensive motivational system to promote behaviours that protect the organism from perceived danger. Normal fear involves a pattern of adaptive responses to conditioned or unconditioned threat stimuli (exteroceptive or interoceptive). Fear can involve internal representations and cognitive processing, and can be modulated by a variety of factors.
Behaviour (RDoC)	Analgesia approach (early development), Avoidance, Facial expressions, Freezing, Open field, Response inhibition, Response time, Risk assessment, Social approach

How important do you think **Acute Threat "Fear"** is to optimal performance in dynamic and high-pressure environments?

- Extremely important
- Very important
- Moderately important
- Slightly important
- Not important / NA
- Don't know / Unsure

Feel free to provide your rationale or reasoning for rating **Acute Threat "Fear"** this way.
Please note that your comments here will be read by the research team and may be presented to all Delphi Panel members unless you select 'HIDDEN' in the following question (in which case only the research team will see the comments). For this reason, please ensure your comments are clear and use language that people outside of your field will understand. You will remain anonymous.

OPTIONAL: Please select 'HIDDEN' if you would like your above response to not be included in the anonymous pool of expert comments at the end of the iteration.

CONSTRUCT	Potential Threat "Anxiety"
Description (RDoC)	Activation of a brain system in which harm may potentially occur but is distant, ambiguous, or low/uncertain in probability, characterized by a pattern of responses such as enhanced risk assessment (vigilance). These responses to low imminence threats are qualitatively different than the high imminence threat behaviours that characterise fear.

How important do you think **Potential Threat "Anxiety"** is to optimal performance in dynamic and high-pressure environments?

- Extremely important
- Very important
- Moderately important
- Slightly important
- Not important / NA
- Don't know / Unsure

Feel free to provide your rationale or reasoning for rating **Potential Threat "Anxiety"** this way.

Please note that your comments here will be read by the research team and may be presented to all Delphi Panel members unless you select 'HIDDEN' in the following question (in which case only the research team will see the comments). For this reason, please ensure your comments are clear and use language that people outside of your field will understand. You will remain anonymous.

OPTIONAL: Please select 'HIDDEN' if you would like your above response to not be included in the anonymous pool of expert comments at the end of the iteration.

CONSTRUCT	Sustained Threat
Description (RDoC)	An aversive emotional state caused by prolonged (i.e., weeks to months) exposure to internal and/or external condition(s), state(s), or stimuli that are adaptive to escape or avoid. The exposure may be actual or anticipated; the changes in affect, cognition, physiology, and behaviour caused by sustained threat persist in the absence of the threat and can be differentiated from those changes evoked by acute threat.
Behaviour (RDoC)	Anhedonia/decreased appetitive behaviour, Anxious Arousal, Attentional bias to threat, Avoidance, Decreased libido, Helplessness behaviour, Increased conflict detection, Increased perseverative behaviour, Memory retrieval deficits, Punishment sensitivity

How important do you think **Sustained Threat** is to optimal performance in dynamic and high-pressure environments?

- Extremely important
- Very important
- Moderately important
- Slightly important
- Not important / NA
- Don't know / Unsure

Feel free to provide your rationale or reasoning for rating **Sustained Threat** this way.
Please note that your comments here will be read by the research team and may be presented to all Delphi Panel members unless you select 'HIDDEN' in the following question (in which case only the research team will see the comments). For this reason, please ensure your comments are clear and use language that people outside of your field will understand. You will remain anonymous.

OPTIONAL: Please select 'HIDDEN' if you would like your above response to not be included in the anonymous pool of expert comments at the end of the iteration.

CONSTRUCT	Loss
Description (RDoC)	A state of deprivation of a motivationally significant con-specific, object, or situation. Loss may be social or non-social and may include permanent or sustained loss of shelter, behavioural control, status, loved ones, or relationships. The response to loss may be episodic (e.g., grief) or sustained.
Behaviour (RDoC)	Amotivation, Anhedonia, Attentional bias to negative valenced information, Crying, Executive function, Guilt, Increased self-focus, Loss of drive, Loss-relevant recall bias, Morbid Thoughts, Psychomotor retardation, Rumination, Sadness, Shame, Withdrawal, Worry

How important do you think **Loss** is to optimal performance in dynamic and high-pressure environments?

- Extremely important
- Very important
- Moderately important
- Slightly important
- Not important / NA
- Don't know / Unsure

Feel free to provide your rationale or reasoning for rating **Loss** this way.
Please note that your comments here will be read by the research team and may be presented to all Delphi Panel members unless you select 'HIDDEN' in the following question (in which case only the research team will see the comments). For this reason, please ensure your comments are clear and use language that people outside of your field will understand. You will remain anonymous.

OPTIONAL: Please select 'HIDDEN' if you would like your above response to not be included in the anonymous pool of expert comments at the end of the iteration.

CONSTRUCT	Frustrative Nonreward
Description (RDoC)	Reactions elicited in response to withdrawal/prevention of reward, i.e., by the inability to obtain positive rewards following repeated or sustained efforts.
Behaviour (RDoC)	Physical and relational aggression

How important do you think **Frustrative Nonreward** is to optimal performance in dynamic and high-pressure environments?

- Extremely important
- Very important
- Moderately important
- Slightly important
- Not important / NA
- Don't know / Unsure

Feel free to provide your rationale or reasoning for rating **Frustrative Nonreward** this way.
Please note that your comments here will be read by the research team and may be presented to all Delphi Panel members unless you select 'HIDDEN' in the following question (in which case only the research team will see the comments). For this reason, please ensure your comments are clear and use language that people outside of your field will understand. You will remain anonymous.

OPTIONAL: Please select 'HIDDEN' if you would like your above response to not be included in the anonymous pool of expert comments at the end of the iteration.

Importance: Positive Valence Systems

DOMAIN: Positive Valence Systems

Description: **Positive Valence Systems** primarily responsible for responses to positive motivational situations or contexts, such as reward seeking, consummatory behavior, and reward/habit learning.

Constructs / Subconstructs

- Construct: Reward Responsiveness
  - Subconstruct: Reward Anticipation
  - Subconstruct: Initial Response to Reward
  - Subconstruct: Reward Satiation
- Construct: Reward Learning
  - Subconstruct: Probabilistic and Reinforcement Learning
  - Subconstruct: Reward Prediction Error
  - Subconstruct: Habit - PVS
- Construct: Reward Valuation
  - Subconstruct: Reward (probability)
  - Subconstruct: Delay
  - Subconstruct: Effort

Definitions of key terms.pdf

The following questions ask you to rate the Subconstructs under the Construct **Reward Responsiveness**

DOMAIN	Positive Valence Systems
**CONSTRUCT**	**Reward Responsiveness**
Description (RDoC)	Processes that govern an organism's hedonic response to impending or possible reward (as reflected in reward anticipation), the receipt of reward (as reflected in initial response to reward) and following repeated receipt of reward (as in reward satiation); across these subdomains, reward responsiveness primarily reflects neural activity to receipt of reward and reward cues and can also be measured in terms of subjective and behavioural responses.
Subconstructs	• Reward Anticipation<br>• Initial Response to Reward<br>• Reward Satiation

CONSTRUCT	Reward Responsiveness
**SUB-CONSTRUCT**	**Reward Anticipation**
Description (RDoC)	Processes associated with the ability to anticipate and/or represent a future incentive—as reflected in language expression, behavioural responses, and/or engagement of the neural systems to cues about a future positive reinforcer.

How important do you think **Reward Anticipation** is to optimal performance in dynamic and high-pressure environments?

- Extremely important
- Very important
- Moderately important
- Slightly important
- Not important / NA
- Don't know / Unsure

Feel free to provide your rationale or reasoning for rating **Reward Anticipation** this way.
Please note that your comments here will be read by the research team and may be presented to all Delphi Panel members unless you select 'HIDDEN' in the following question (in which case only the research team will see the comments). For this reason, please ensure your comments are clear and use language that people outside of your field will understand. You will remain anonymous.

OPTIONAL: Please select 'HIDDEN' if you would like your above response to not be included in the anonymous pool of expert comments at the end of the iteration.

CONSTRUCT	Reward Responsiveness
**SUB-CONSTRUCT**	**Initial Response to Reward**
Description (RDoC)	Processes evoked by the initial presentation of a positive reinforcer as reflected by indices of neuronal activity and verbal or behavioural responses.
Behaviour (RDoC)	Taste reactivity

How important do you think **Initial Response to Reward** is to optimal performance in dynamic and high-pressure environments?

- Extremely important
- Very important
- Moderately important

- Slightly important
- Not important / NA
- Don't know / Unsure

Feel free to provide your rationale or reasoning for rating **Initial Response to Reward** this way.
Please note that your comments here will be read by the research team and may be presented to all Delphi Panel members unless you select 'HIDDEN' in the following question (in which case only the research team will see the comments). For this reason, please ensure your comments are clear and use language that people outside of your field will understand. You will remain anonymous.

OPTIONAL: Please select 'HIDDEN' if you would like your above response to not be included in the anonymous pool of expert comments at the end of the iteration.

CONSTRUCT	Reward Responsiveness
**SUB-CONSTRUCT**	**Reward Satiation**
Description (RDoC)	Processes associated with the change in incentive value of a reinforcer over time as that reinforcer is consumed or experienced, as reflected in language expression, behavioural responses, and/or engagement of the neural systems.

How important do you think **Reward Satiation** is to optimal performance in dynamic and high-pressure environments?

- Extremely important
- Very important
- Moderately important
- Slightly important
- Not important / NA
- Don't know / Unsure

Feel free to provide your rationale or reasoning for rating **Reward Satiation** this way.
Please note that your comments here will be read by the research team and may be presented to all Delphi Panel members unless you select 'HIDDEN' in the following question (in which case only the research team will see the

comments). For this reason, please ensure your comments are clear and use language that people outside of your field will understand. You will remain anonymous.

OPTIONAL: Please select 'HIDDEN' if you would like your above response to not be included in the anonymous pool of expert comments at the end of the iteration.

The following questions ask you to rate the Subconstructs under the Construct **Reward Learning**

DOMAIN	Positive Valence Systems
**CONSTRUCT**	**Reward Learning**
Description (RDoC)	A process by which organisms acquire information about stimuli, actions, and contexts that predict positive outcomes, and by which behaviour is modified when a novel reward occurs, or outcomes are better than expected. Reward learning is a type of reinforcement learning.
Subconstructs	• Probabilistic and Reinforcement Learning<br>• Reward Prediction Error<br>• Habit – PVS

CONSTRUCT	Reward Learning
**SUB-CONSTRUCT**	**Probabilistic and Reinforcement Learning**
Description (RDoC)	The ability to learn which actions or stimuli are associated with obtaining a reinforcer, even when a particular action or stimulus is not always associated with obtaining the reinforcer.

How important do you think **Probabilistic and Reinforcement Learning** is to optimal performance in dynamic and high-pressure environments?

- Extremely important
- Very important
- Moderately important
- Slightly important
- Not important / NA

○ Don't know / Unsure

Feel free to provide your rationale or reasoning for rating **Probabilistic and Reinforcement Learning** this way.

Please note that your comments here will be read by the research team and may be presented to all Delphi Panel members unless you select 'HIDDEN' in the following question (in which case only the research team will see the comments). For this reason, please ensure your comments are clear and use language that people outside of your field will understand. You will remain anonymous.

OPTIONAL: Please select 'HIDDEN' if you would like your above response to not be included in the anonymous pool of expert comments at the end of the iteration.

CONSTRUCT	Reward Learning
**SUB-CONSTRUCT**	**Reward Prediction Error**
Description (RDoC)	Processes associated with the difference between anticipated and obtained rewards are important for reinforcement learning. The error can indicate that the reward received was either larger than expected (positive prediction error) or smaller than expected (negative prediction error).
Behaviour (RDoC)	Goal tracking, Pavlovian approach, Reward-related speeding, Sign tracking

How important do you think **Reward Prediction Error** is to optimal performance in dynamic and high-pressure environments?

○ Extremely important
○ Very important
○ Moderately important
○ Slightly important
○ Not important / NA
○ Don't know / Unsure

Feel free to provide your rationale or reasoning for rating **Reward Prediction Error** this way.

Please note that your comments here will be read by the research team and may be presented to all Delphi Panel members unless you select 'HIDDEN' in the following question (in which case only the research team will see the

comments). For this reason, please ensure your comments are clear and use language that people outside of your field will understand. You will remain anonymous.

OPTIONAL: Please select 'HIDDEN' if you would like your above response to not be included in the anonymous pool of expert comments at the end of the iteration.

CONSTRUCT	Reward Learning
**SUB-CONSTRUCT**	**Habit**
Description (RDoC)	Sequential, repetitive, motor behaviours or cognitive processes elicited by external or internal triggers that, once initiated, can go to completion without continuous effortful oversight. Habits can be adaptive by virtue of freeing up cognitive resources. Habit formation is a frequent consequence of reward learning, but, over time, its expression can become resistant to changes in outcome value. Some habit-related behaviours could be pathological expressions of processes that under other circumstances subserve adaptive goals.
Behaviour (RDoC)	Compulsive behaviours, Repetitive behaviours, Stereotypic behaviours

How important do you think **Habit** is to optimal performance in dynamic and high-pressure environments?

- Extremely important
- Very important
- Moderately important
- Slightly important
- Not important / NA
- Don't know / Unsure

Feel free to provide your rationale or reasoning for rating **Habit** this way.

Please note that your comments here will be read by the research team and may be presented to all Delphi Panel members unless you select 'HIDDEN' in the following question (in which case only the research team will see the comments). For this reason, please ensure your comments are clear and use language that people outside of your field will understand. You will remain anonymous.

OPTIONAL: Please select 'HIDDEN' if you would like your above response to not be included in the anonymous pool of expert comments at the end of the iteration.

The following questions ask you to rate the Subconstructs under the Construct **Reward Valuation**

DOMAIN	Positive Valence Systems
**CONSTRUCT**	**Reward Valuation**
Description (RDoC)	Processes by which the probability and benefits of a prospective outcome are computed by reference to external information, social context (e.g., group input), and/or prior experience. This computation is influenced by pre-existing biases, learning, memory, stimulus characteristics, and deprivation states. Reward valuation may involve the assignment of incentive salience to stimuli.
Subconstructs	• Reward (probability)<br>• Delay<br>• Effort

CONSTRUCT	Reward Valuation
**SUB-CONSTRUCT**	**Reward (probability)**
Description (RDoC)	Process by which the value of a reinforcer is computed as a function of its magnitude, valence, and predictability.

How important do you think **Reward (Probability)** is to optimal performance in dynamic and high-pressure environments?

- Extremely important
- Very important
- Moderately important
- Slightly important
- Not important / NA
- Don't know / Unsure

Feel free to provide your rationale or reasoning for rating **Reward (Probability)** this way.
Please note that your comments here will be read by the research team and may be presented to all Delphi Panel members unless you select 'HIDDEN' in the following question (in which case only the research team will see the comments). For this reason, please ensure your comments are clear and use language that people outside of your field will understand. You will remain anonymous.

OPTIONAL: Please select 'HIDDEN' if you would like your above response to not be included in the anonymous pool of expert comments at the end of the iteration.

CONSTRUCT	Reward Valuation
**SUB-CONSTRUCT**	**Delay**
Description (RDoC)	Processes by which the value of a reinforcer is computed as a function of its magnitude and the time interval prior to its expected delivery.

How important do you think **Delay** is to optimal performance in dynamic and high-pressure environments?

- Extremely important
- Very important
- Moderately important
- Slightly important
- Not important / NA
- Don't know / Unsure

Feel free to provide your rationale or reasoning for rating **Delay** this way.
Please note that your comments here will be read by the research team and may be presented to all Delphi Panel members unless you select 'HIDDEN' in the following question (in which case only the research team will see the comments). For this reason, please ensure your comments are clear and use language that people outside of your field will understand. You will remain anonymous.

OPTIONAL: Please select 'HIDDEN' if you would like your above response to not be included in the anonymous pool of expert comments at the end of the iteration.

CONSTRUCT	Reward Valuation
**SUB-CONSTRUCT**	**Effort**
Description (RDoC)	Processes by which the value of a reinforcer is computed as a function of its magnitude and the perceived costs of the physical or cognitive effort required to obtain it.

How important do you think **Effort** is to optimal performance in dynamic and high-pressure environments?

- Extremely important
- Very important
- Moderately important
- Slightly important
- Not important / NA
- Don't know / Unsure

Feel free to provide your rationale or reasoning for rating **Effort** this way.
Please note that your comments here will be read by the research team and may be presented to all Delphi Panel members unless you select 'HIDDEN' in the following question (in which case only the research team will see the comments). For this reason, please ensure your comments are clear and use language that people outside of your field will understand. You will remain anonymous.

OPTIONAL: Please select 'HIDDEN' if you would like your above response to not be included in the anonymous pool of expert comments at the end of the iteration.

Importance: Cognitive Systems

DOMAIN: Cognitive Systems

Description: **Cognitive Systems** are responsible for various cognitive processes.

Constructs/Subconstructs

- Construct: Attention
- Construct: Perception
    - Subconstruct: Visual Perception
    - Subconstruct: Auditory Perception
    - Subconstruct: Olfactory/Somatosensory/Multimodal/Perception
- Construct: Declarative Memory
- Construct: Language
- Construct: Cognitive Control
    - Subconstruct: Goal Selection; Updating, Representation, and Maintenance
    - Subconstruct: Response Selection; Inhibition/Suppression
    - Subconstruct: Performance Monitoring
- Construct: Working Memory
    - Subconstruct: Active Maintenance
    - Subconstruct: Flexible Updating
    - Subconstruct: Limited Capacity
    - Subconstruct: Interference Control

Definitions of key terms.pdf

CONSTRUCT	Attention
Description (RDoC)	Attention refers to a range of processes that regulate access to capacity-limited systems, such as awareness, higher perceptual processes, and motor action. The concepts of capacity limitation and competition are inherent to the concepts of selective and divided attention.
Behaviour (RDoC)	ANT task distractibility, Attentional lapses vs sustained attention, Distractibility, Object/feature attention, Psychophysics, Spatial attention

How important do you think **Attention** is to optimal performance in dynamic and high-pressure environments?

○ Extremely important

- Very important
- Moderately important
- Slightly important
- Not important / NA
- Don't know / Unsure

Feel free to provide your rationale or reasoning for rating **Attention** this way.
Please note that your comments here will be read by the research team and may be presented to all Delphi Panel members unless you select 'HIDDEN' in the following question (in which case only the research team will see the comments). For this reason, please ensure your comments are clear and use language that people outside of your field will understand. You will remain anonymous.

OPTIONAL: Please select 'HIDDEN' if you would like your above response to not be included in the anonymous pool of expert comments at the end of the iteration.

DOMAIN	Cognitive Systems
**CONSTRUCT**	**Perception**
Description (RDoC)	Perception refers to the process(es) that perform computations on sensory data to construct and transform representations of the external environment, acquire information from, and make predictions about, the external world, and guide action.
Subconstructs	• Visual Perception<br>• Auditory Perception<br>• Olfactory/Somatosensory/Multimodal/Perception

The following questions ask you to rate the Subconstructs under the Construct **Perception**

CONSTRUCT	Perception
**SUB-CONSTRUCT**	**Visual Perception**
Description (Delphi Team)	Refers to the process(es) that perform computations on sensory data to construct and transform representations of the external environment, acquire information from, and make predictions about, the external world, and guide action.
Behaviour (RDoC)	Discrimination, identification and localization, Perceptual learning, Perceptual priming, Reading, Stimulus detection, Visual acuity

How important do you think **Visual Perception** is to optimal performance in dynamic and high-pressure environments?

- Extremely important
- Very important
- Moderately important
- Slightly important
- Not important / NA
- Don't know / Unsure

Feel free to provide your rationale or reasoning for rating **Visual Perception** this way.
Please note that your comments here will be read by the research team and may be presented to all Delphi Panel members unless you select 'HIDDEN' in the following question (in which case only the research team will see the comments). For this reason, please ensure your comments are clear and use language that people outside of your field will understand. You will remain anonymous.

OPTIONAL: Please select 'HIDDEN' if you would like your above response to not be included in the anonymous pool of expert comments at the end of the iteration.

CONSTRUCT	Perception
**SUB-CONSTRUCT**	**Auditory Perception**
Description (Delphi Team)	Refers to the process(es) that perform computations on auditory data to construct and transform representations of the external environment, acquire information from, and make predictions about, the external world, and guide action.
Behaviour (RDoC)	Perceptual identification, Perceptual learning, Perceptual priming, Spatial localization, Stimulus detection

How important do you think **Auditory Perception** is to optimal performance in dynamic and high-pressure environments?

- Extremely important
- Very important

- Moderately important
- Slightly important
- Not important / NA
- Don't know / Unsure

Feel free to provide your rationale or reasoning for rating **Auditory Perception** this way.
Please note that your comments here will be read by the research team and may be presented to all Delphi Panel members unless you select 'HIDDEN' in the following question (in which case only the research team will see the comments). For this reason, please ensure your comments are clear and use language that people outside of your field will understand. You will remain anonymous.

OPTIONAL: Please select 'HIDDEN' if you would like your above response to not be included in the anonymous pool of expert comments at the end of the iteration.

CONSTRUCT	Perception
**SUB-CONSTRUCT**	**Olfactory/Somatosensory/Multimodal/Perception**
Description (Delphi Team)	Refers to the process(es) that perform computations on olfactory/ somatosensory/multimodal/perception data to construct and transform representations of the external environment, acquire information from, and make predictions about, the external world, and guide action.

How important do you think **Olfactory/Somatosensory/Multimodal/Perception** is to optimal performance in dynamic and high-pressure environments?

- Extremely important
- Very important
- Moderately important
- Slightly important
- Not important / NA
- Don't know / Unsure

Feel free to provide your rationale or reasoning for rating **Olfactory/Somatosensory/Multimodal/Perception** this way.

Please note that your comments here will be read by the research team and may be presented to all Delphi Panel members unless you select 'HIDDEN' in the following question (in which case only the research team will see the comments). For this reason, please ensure your comments are clear and use language that people outside of your field will understand. You will remain anonymous.

OPTIONAL: Please select 'HIDDEN' if you would like your above response to not be included in the anonymous pool of expert comments at the end of the iteration.

CONSTRUCT	Declarative Memory
Description (RDoC)	Declarative memory is the acquisition or encoding, storage and consolidation, and retrieval of representations of facts and events. Declarative memory provides the critical substrate for relational representations—i.e., for spatial, temporal, and other contextual relations among items, contributing to representations of events (episodic memory) and the integration and organization of factual knowledge (semantic memory). These representations facilitate the inferential and flexible extraction of new information from these relationships.
Behaviour (RDoC)	Discrimination, Familiarity, Learning, Recall, Recognition

How important do you think **Declarative Memory** is to optimal performance in dynamic and high-pressure environments?

- Extremely important
- Very important
- Moderately important
- Slightly important
- Not important / NA
- Don't know / Unsure

Feel free to provide your rationale or reasoning for rating **Declarative Memory** this way.

Please note that your comments here will be read by the research team and may be presented to all Delphi Panel members unless you select 'HIDDEN' in the following question (in which case only the research team will see the

comments). For this reason, please ensure your comments are clear and use language that people outside of your field will understand. You will remain anonymous.

OPTIONAL: Please select 'HIDDEN' if you would like your above response to not be included in the anonymous pool of expert comments at the end of the iteration.

CONSTRUCT	Language
Description (RDoC)	Language is a system of shared symbolic representations of the world, the self and abstract concepts that supports thought and communication.
Behaviour (RDoC)	Coherent discourse, Coherent sentences, Production and comprehension of words

How important do you think **Language** is to optimal performance in dynamic and high-pressure environments?

- Extremely important
- Very important
- Moderately important
- Slightly important
- Not important / NA
- Don't know / Unsure

Feel free to provide your rationale or reasoning for rating **Language** this way.

Please note that your comments here will be read by the research team and may be presented to all Delphi Panel members unless you select 'HIDDEN' in the following question. For this reason, please ensure your comments are clear and use language that people outside of your field will understand. You will remain anonymous.

OPTIONAL: Please select 'HIDDEN' if you would like your above response to not be included in the anonymous pool of expert comments at the end of the iteration.

The following questions ask you to rate the Subconstructs under the Construct **Cognitive Control**

DOMAIN	Cognitive Systems
**CONSTRUCT**	**Cognitive Control**
Description (RDoC)	A system that modulates the operation of other cognitive and emotional systems, in the service of goal-directed behaviour, when prepotent modes of responding are not adequate to meet the demands of the current context. Additionally, control processes are engaged in the case of novel contexts, where appropriate responses need to be selected from among competing alternatives.
Subconstructs	• Goal Selection, Updating, Representation, and Maintenance<br>• Response Selection; Inhibition/Suppression<br>• Performance Monitoring

How important do you think **Goal Selection; Updating, Representation, and Maintenance** is to optimal performance in dynamic and high-pressure environments?

- Extremely important
- Very important
- Moderately important
- Slightly important
- Not important / NA
- Don't know / Unsure

Feel free to provide your rationale or reasoning for rating **Goal Selection; Updating, Representation, and Maintenance** this way.

Please note that your comments here will be read by the research team and may be presented to all Delphi Panel members unless you select 'HIDDEN' in the following question (in which case only the research team will see the comments). For this reason, please ensure your comments are clear and use language that people outside of your field will understand. You will remain anonymous.

OPTIONAL: Please select 'HIDDEN' if you would like your above response to not be included in the anonymous pool of expert comments at the end of the iteration.

CONSTRUCT	Cognitive Control
**SUB-CONSTRUCT**	**Response Selection; Inhibition/Suppression**
Description (Delphi Team)	The ability to select, inhibit and supress responses, particularly in novel situations where appropriate responses need to be selected amongst competing alternatives.
Behaviour (RDoC)	Distractibility, Impulsive behaviours, Off-task behaviours

How important do you think **Response Selection; Inhibition/Suppression** is to optimal performance in dynamic and high-pressure environments?

- Extremely important
- Very important
- Moderately important
- Slightly important
- Not important / NA
- Don't know / Unsure

Feel free to provide your rationale or reasoning for rating **Response Selection; Inhibition/Suppression** this way.

Please note that your comments here will be read by the research team and may be presented to all Delphi Panel members unless you select 'HIDDEN' in the following question (in which case only the research team will see the comments). For this reason, please ensure your comments are clear and use language that people outside of your field will understand. You will remain anonymous.

OPTIONAL: Please select 'HIDDEN' if you would like your above response to not be included in the anonymous pool of expert comments at the end of the iteration.

How important do you think **Performance Monitoring** is to optimal performance in dynamic and high-pressure environments?

- Extremely important
- Very important
- Moderately important
- Slightly important
- Not important / NA
- Don't know / Unsure

Feel free to provide your rationale or reasoning for rating **Performance Monitoring** this way.
Please note that your comments here will be read by the research team and may be presented to all Delphi Panel members unless you select 'HIDDEN' in the following question (in which case only the research team will see the comments). For this reason, please ensure your comments are clear and use language that people outside of your field will understand. You will remain anonymous.

OPTIONAL: Please select 'HIDDEN' if you would like your above response to not be included in the anonymous pool of expert comments at the end of the iteration.

The following questions ask you to rate the Subconstructs under the Construct **Working Memory**

DOMAIN	Cognitive Systems
**CONSTRUCT**	**Working Memory**
Description (RDoC)	Working Memory is the active maintenance and flexible updating of goal/task relevant information (items, goals, strategies, etc.) in a form that has limited capacity and resists interference. These representations: may involve flexible binding of representations; may be characterized by the absence of external support for the internally maintained representations; and are frequently temporary, though this may be due to ongoing interference. It involves active maintenance, flexible updating, limited capacity, and interference control.
Subconstructs	• Active Maintenance<br>• Flexible Updating<br>• Limited Capacity<br>• Interference Control

CONSTRUCT	Working Memory
**SUB-CONSTRUCT**	**Active Maintenance**
Description (Delphi Team)	The ability to actively maintain one or more pieces of information as internal representations, which activates brain regions that are specific to the modality of the information being maintained. Active maintenance is one of the features which distinguished working memory from other cognitive processes.

How important do you think **Active Maintenance** is to optimal performance in dynamic and high-pressure environments?

- Extremely important
- Very important
- Moderately important
- Slightly important
- Not important / NA
- Don't know / Unsure

Feel free to provide your rationale or reasoning for rating **Active Maintenance** this way.

Please note that your comments here will be read by the research team and may be presented to all Delphi Panel members unless you select 'HIDDEN' in the following question (in which case only the research team will see the comments). For this reason, please ensure your comments are clear and use language that people outside of your field will understand. You will remain anonymous.

OPTIONAL: Please select 'HIDDEN' if you would like your above response to not be included in the anonymous pool of expert comments at the end of the iteration.

CONSTRUCT	Working Memory
**SUB-CONSTRUCT**	**Flexible Updating**
Description (Delphi Team)	The ability to change and update goal/task relevant information (items, goals, strategies, etc.) in accordance with the task at hand.

How important do you think **Flexible Updating** is to optimal performance in dynamic and high-pressure environments?

- Extremely important
- Very important
- Moderately important
- Slightly important
- Not important / NA
- Don't know / Unsure

Feel free to provide your rationale or reasoning for rating **Flexible Updating** this way.
Please note that your comments here will be read by the research team and may be presented to all Delphi Panel members unless you select 'HIDDEN' in the following question (in which case only the research team will see the comments). For this reason, please ensure your comments are clear and use language that people outside of your field will understand. You will remain anonymous.

OPTIONAL: Please select 'HIDDEN' if you would like your above response to not be included in the anonymous pool of expert comments at the end of the iteration.

CONSTRUCT	Working Memory
**SUB-CONSTRUCT**	**Limited Capacity**
Description (Delphi Team)	Reflect a major component of working memory impairment in many forms of psychopathology.

How important do you think **Limited Capacity** is to optimal performance in dynamic and high-pressure environments?

- Extremely important
- Very important
- Moderately important
- Slightly important
- Not important / NA
- Don't know / Unsure

Feel free to provide your rationale or reasoning for rating **Limited Capacity** this way.
Please note that your comments here will be read by the research team and may be presented to all Delphi Panel members unless you select 'HIDDEN' in the following question (in which case only the research team will see the comments). For this reason, please ensure your comments are clear and use language that people outside of your field will understand. You will remain anonymous.

OPTIONAL: Please select 'HIDDEN' if you would like your above response to not be included in the anonymous pool of expert comments at the end of the iteration.

CONSTRUCT	Working Memory
**SUB-CONSTRUCT**	**Interference Control**
Description (Delphi Team)	The ability to maintain focus and stay on task whilst resisting interference. The ability to resist interference is made more difficult by behavioural data from a secondary task that uses the same modality or type of information being maintained in working memory. Many working memory tasks do not involve specific manipulations of interference, although it is often assumed that interference in always occurring via the influence of previous stimulus traces, stimulus response mappings, or other information in the environment. Interference control can be tested be the explicit presentation of competing information, goals or tasks.

How important do you think **Interference Control** is to optimal performance in dynamic and high-pressure environments?

- Extremely important
- Very important

- Moderately important
- Slightly important
- Not important / NA
- Don't know / Unsure

Feel free to provide your rationale or reasoning for rating **Interference Control** this way.
Please note that your comments here will be read by the research team and may be presented to all Delphi Panel members unless you select 'HIDDEN' in the following question (in which case only the research team will see the comments). For this reason, please ensure your comments are clear and use language that people outside of your field will understand. You will remain anonymous.

OPTIONAL: Please select 'HIDDEN' if you would like your above response to not be included in the anonymous pool of expert comments at the end of the iteration.

Importance: Social Processes

DOMAIN: Social Processes

Description: **Systems for Social Processes** mediate responses to interpersonal settings of various types, including perception and interpretation of others' actions.

Constructs/Subconstructs

- Construct: Affiliation and Attachment
- Construct: Social Communication
  - Subconstruct: Reception of Facial Communication
  - Subconstruct: Production of Facial Communication
  - Subconstruct: Reception of Non-Facial Communication
  - Subconstruct: Production of Non-Facial Communication
- Construct: Perception and Understanding of Self
  - Subconstruct: Agency
  - Subconstruct: Self-Knowledge
- Construct: Perception and Understanding of Others

- Subconstruct: Animacy Perception
- Subconstruct: Action Perception
- Subconstruct: Understanding Mental States

Definitions of key terms.pdf

CONSTRUCT	Affiliation and Attachment
Description (RDoC)	Affiliation is engagement in positive social interactions with other individuals. Attachment is selective affiliation as a consequence of the development of a social bond. Affiliation and Attachment are moderated by social information processing (processing of social cues) and social motivation. Affiliation is a behavioural consequence of social motivation and can manifest itself in social approach behaviours. Affiliation and Attachment require detection of and attention to social cues, as well as social learning and memory associated with the formation of relationships. Affiliation and Attachment include both the positive physiological consequences of social interactions and the behavioural and physiological consequences of disruptions to social relationships. Clinical manifestations of disruptions in Affiliation and Attachment include social withdrawal, social indifference and anhedonia, and over-attachment.
Behaviour (RDoC)	Attachment Formation: Maintaining proximity, Preference for individual<br>Attachment Maintenance: Distress upon separation

How important do you think **Affiliation and Attachment** is to optimal performance in dynamic and high-pressure environments?

- Extremely important
- Very important
- Moderately important
- Slightly important
- Not important / NA
- Don't know / Unsure

Feel free to provide your rationale or reasoning for rating **Affiliation and Attachment** this way.

Please note that your comments here will be read by the research team and may be presented to all Delphi Panel members unless you select 'HIDDEN' in the following question (in which case only the research team will see the comments). For this reason, please ensure your comments are clear and use language that people outside of your field will understand. You will remain anonymous.

OPTIONAL: Please select 'HIDDEN' if you would like your above response to not be included in the anonymous pool of expert comments at the end of the iteration.

The following questions ask you to rate the Subconstructs under the Construct **Social Communication**

DOMAIN	Social Processes
**CONSTRUCT**	**Social Communication**
Description (RDoC)	A dynamic process that includes both receptive and productive aspects used for exchange of socially relevant information. Social communication is essential for the integration and maintenance of the individual in the social environment. This Construct is reciprocal and interactive, and social communication abilities may appear very early in life. Social communication is distinguishable from other cognitive systems (e.g., perception, cognitive control, memory, attention) in that it particularly involves interactions with conspecifics. The underlying neural substrates of social communication evolved to support both automatic/reflexive and volitional control, including the motivation and ability to engage in social communication. Receptive aspects may be implicit or explicit; examples include affect recognition, facial recognition and characterization. Productive aspects include eye contact, expressive reciprocation, and gaze following. Although facial communication was set aside as a separate sub-construct for the purposes of identifying matrix elements, social communication typically utilizes information from several modalities, including facial, vocal, gestural, postural, and olfactory processing.
Subconstructs	• Reception of Facial Communication<br>• Production of Facial Communication<br>• Reception of Non-Facial Communication<br>• Production of Non-Facial Communication

CONSTRUCT	Social Communication
**SUB-CONSTRUCT**	**Reception of Facial Communication**
Description (RDoC)	The capacity to perceive someone's emotional state non-verbally based on facial expressions.
Behaviour (RDoC)	Behavioural observation/coding systems, Eye gaze detection, Identification of emotion, Implicit mimicry, Scanning patterns

How important do you think **Reception of Facial Communication** is to optimal performance in dynamic and high-pressure environments?

- Extremely important
- Very important
- Moderately important
- Slightly important
- Not important / NA
- Don't know / Unsure

Feel free to provide your rationale or reasoning for rating **Reception of Facial Communication** this way.

Please note that your comments here will be read by the research team and may be presented to all Delphi Panel members unless you select 'HIDDEN' in the following question (in which case only the research team will see the comments). For this reason, please ensure your comments are clear and use language that people outside of your field will understand. You will remain anonymous.

OPTIONAL: Please select 'HIDDEN' if you would like your above response to not be included in the anonymous pool of expert comments at the end of the iteration.

CONSTRUCT	Social Communication
**SUB-CONSTRUCT**	**Production of Facial Communication**
Description (RDoC)	The capacity to convey one's emotional state non-verbally via facial expression.
Behaviour (RDoC)	Behavioural observation/coding systems, Eye gaze aversion/contact, Facial affect production, Head turning, Imitation of facial gestures, Joint attention, Reciprocal emotional expression, Reciprocal eye contact

How important do you think **Production of Facial Communication** is to optimal performance in dynamic and high-pressure environments?

- Extremely important
- Very important
- Moderately important

- Slightly important
- Not important / NA
- Don't know / Unsure

Feel free to provide your rationale or reasoning for rating **Production of Facial Communication** this way.

Please note that your comments here will be read by the research team and may be presented to all Delphi Panel members unless you select 'HIDDEN' in the following question (in which case only the research team will see the comments). For this reason, please ensure your comments are clear and use language that people outside of your field will understand. You will remain anonymous.

OPTIONAL: Please select 'HIDDEN' if you would like your above response to not be included in the anonymous pool of expert comments at the end of the iteration.

CONSTRUCT	Social Communication
**SUB-CONSTRUCT**	**Reception of Non-Facial Communication**
Description (RDoC)	The capacity to perceive social and emotional information based on modalities other than facial expression, including non-verbal gestures, affective prosody, distress calling, cooing, etc.
Behaviour (RDoC)	Comprehension of emotional prosody, Comprehension of non-verbal gestures, Humour comprehension, Irony/sarcasm comprehension, Metaphor comprehension

How important do you think **Reception of Non-Facial Communication** is to optimal performance in dynamic and high-pressure environments?

- Extremely important
- Very important
- Moderately important
- Slightly important
- Not important / NA
- Don't know / Unsure

Feel free to provide your rationale or reasoning for rating **Reception of Non-Facial Communication** this way.

Please note that your comments here will be read by the research team and may be presented to all Delphi Panel members unless you select 'HIDDEN' in the following question (in which case only the research team will see the comments). For this reason, please ensure your comments are clear and use language that people outside of your field will understand. You will remain anonymous.

OPTIONAL: Please select 'HIDDEN' if you would like your above response to not be included in the anonymous pool of expert comments at the end of the iteration.

CONSTRUCT	Social Communication
**SUB-CONSTRUCT**	**Production of Non-Facial Communication**
Description (RDoC)	The capacity to express social and emotional information based on modalities other than facial expression, including non-verbal gestures, affective prosody, distress calling, cooing, etc.
Behaviour (RDoC)	Crying/laughing, Gestural/postural expressions, Interactive play, Response to distress/separation distress, Speech (affective) prosody, Vocalizations

How important do you think **Production of Non-Facial Communication** is to optimal performance in dynamic and high-pressure environments?

- Extremely important
- Very important
- Moderately important
- Slightly important
- Not important / NA
- Don't know / Unsure

Feel free to provide your rationale or reasoning for rating **Production of Non-Facial Communication** this way.

Please note that your comments here will be read by the research team and may be presented to all Delphi Panel members unless you select 'HIDDEN' in the following question (in which case only the research team will see the comments). For this reason, please ensure your comments are clear and use language that people outside of your field will understand. You will remain anonymous.

OPTIONAL: Please select 'HIDDEN' if you would like your above response to not be included in the anonymous pool of expert comments at the end of the iteration.

The following questions ask you to rate the Subconstructs under the Construct **Perception and Understanding of Self**

DOMAIN	Social Processes
**CONSTRUCT**	**Perception and Understanding of Self**
Description (RDoC)	The processes and/or representations involved in being aware of, accessing knowledge about, and/or making judgments about the self. These processes/representations can include current cognitive or emotional internal states, traits, and/or abilities, either in isolation or in relationship to others, as well as the mechanisms that support self-awareness, self-monitoring, and self-knowledge.
Subconstructs	• Agency<br>• Self-Knowledge

CONSTRUCT	Perception and Understanding of Self
**SUB-CONSTRUCT**	**Agency**
Description (RDoC)	The ability to recognize one's self as the agent of one's actions and thoughts, including the recognition of one's own body/body parts.
Behaviour (RDoC)	Delusions of control, Evidence that one understands ownership of one's own body parts or action (thoughts/behaviours), Hallucinations, Stereotypic behaviours.

How important do you think **Agency** is to optimal performance in dynamic and high-pressure environments?

- Extremely important
- Very important
- Moderately important
- Slightly important
- Not important / NA
- Don't know / Unsure

Feel free to provide your rationale or reasoning for rating **Agency** this way.
Please note that your comments here will be read by the research team and may be presented to all Delphi Panel members unless you select 'HIDDEN' in the following question (in which case only the research team will see the comments). For this reason, please ensure your comments are clear and use language that people outside of your field will understand. You will remain anonymous.

OPTIONAL: Please select 'HIDDEN' if you would like your above response to not be included in the anonymous pool of expert comments at the end of the iteration.

CONSTRUCT	Perception and Understanding of Self
**SUB-CONSTRUCT**	**Self-Knowledge**
Description (RDoC)	The ability to make judgments about one's current cognitive or emotional internal states, traits, and/or abilities.
Behaviour (RDoC)	Developmentally appropriate perception of one's competences, skills, abilities beliefs, intentions, desires, and/or emotional states

How important do you think **Self knowledge** is to optimal performance in dynamic and high-pressure environments?

- Extremely important
- Very important
- Moderately important
- Slightly important
- Not important / NA
- Don't know / Unsure

Feel free to provide your rationale or reasoning for rating **Self Knowledge** this way.
Please note that your comments here will be read by the research team and may be presented to all Delphi Panel members unless you select 'HIDDEN' in the following question (in which case only the research team will see the comments). For this reason, please ensure your comments are clear and use language that people outside of your field will understand. You will remain anonymous.

OPTIONAL: Please select 'HIDDEN' if you would like your above response to not be included in the anonymous pool of expert comments at the end of the iteration.

The following questions ask you to rate the Subconstructs under the Construct **Perception and Understanding of Others**

DOMAIN	Social Processes
**CONSTRUCT**	**Perception and Understanding of Others**
Description (RDoC)	The processes and/or representations involved in being aware of, accessing knowledge about, reasoning about, and/or making judgments about other animate entities, including information about cognitive or emotional states, traits or abilities.
Subconstructs	• Animacy Perception<br>• Action Perception<br>• Understanding Mental States

CONSTRUCT	Perception and Understanding of Others
**SUB-CONSTRUCT**	**Animacy Perception**
Description (RDoC)	The ability to appropriately perceive that another entity is an agent (i.e., has a face, interacts contingently, and exhibits biological motion).
Behaviour (RDoC)	Ability to appropriately attribute animacy to other agents

How important do you think **Animacy Perception** is to optimal performance in dynamic and high-pressure environments?

- Extremely important
- Very important
- Moderately important
- Slightly important
- Not important / NA
- Don't know / Unsure

Feel free to provide your rationale or reasoning for rating **Animacy Perception** this way.

Please note that your comments here will be read by the research team and may be presented to all Delphi Panel members unless you select 'HIDDEN' in the following question (in which case only the research team will see the comments). For this reason, please ensure your comments are clear and use language that people outside of your field will understand. You will remain anonymous.

OPTIONAL: Please select 'HIDDEN' if you would like your above response to not be included in the anonymous pool of expert comments at the end of the iteration.

CONSTRUCT	Perception and Understanding of Others
**SUB-CONSTRUCT**	**Action Perception**
Description (Delphi Team)	The ability to perceive the purpose of an action being performed by an animate entity.
Behaviour (RDoC)	Ability to identify what actions an agent is executing, Gaze following, Imitation, Mimicry

How important do you think **Action Perception** is to optimal performance in dynamic and high-pressure environments?

- Extremely important
- Very important
- Moderately important
- Slightly important
- Not important / NA
- Don't know / Unsure

Feel free to provide your rationale or reasoning for rating **Action Perception** this way.

Please note that your comments here will be read by the research team and may be presented to all Delphi Panel members unless you select 'HIDDEN' in the following question (in which case only the research team will see the comments). For this reason, please ensure your comments are clear and use language that people outside of your field will understand. You will remain anonymous.

OPTIONAL: Please select 'HIDDEN' if you would like your above response to not be included in the anonymous pool of expert comments at the end of the iteration.

CONSTRUCT	Perception and Understanding of Others
**SUB-CONSTRUCT**	**Understanding Mental States**
Description (RDoC)	The ability to make judgments and/or attributions about the mental state of other animate entities that allows one to predict or interpret their behaviours. Mental state refers to intentions, beliefs, desires, and emotions.
Behaviour (RDoC)	Developmentally appropriate interpretations of other intentions, goals and beliefs

How important do you think **Understanding Mental States** is to optimal performance in dynamic and high-pressure environments?

- Extremely important
- Very important
- Moderately important
- Slightly important
- Not important / NA
- Don't know / Unsure

Feel free to provide your rationale or reasoning for rating **Understanding Mental States** this way.

Please note that your comments here will be read by the research team and may be presented to all Delphi Panel members unless you select 'HIDDEN' in the following question (in which case only the research team will see the comments). For this reason, please ensure your comments are clear and use language that people outside of your field will understand. You will remain anonymous.

OPTIONAL: Please select 'HIDDEN' if you would like your above response to not be included in the anonymous pool of expert comments at the end of the iteration.

Importance: Arousal and Regulatory Systems

DOMAIN: Arousal and Regulatory Systems

Description: **Arousal/Regulatory Systems** are responsible for generating activation of neural systems as appropriate for various contexts, and providing appropriate homeostatic regulation of such systems as energy balance and sleep.

Constructs/Subconstructs

- Construct: Arousal
- Construct: Circadian Rhythms
- Construct: Sleep-Wakefulness

Definitions of key terms.pdf

CONSTRUCT	Arousal
Description (RDoC)	Arousal is a continuum of sensitivity of the organism to stimuli, both external and internal. Arousal:<br>1. Facilitates interaction with the environment in a context-specific manner (e.g., under conditions of threat, some stimuli must be ignored while sensitivity to and responses to others is enhanced, as exemplified in the startle reflex),<br>2. Can be evoked by either external/environmental stimuli or internal stimuli (e.g., emotions and cognition),<br>3. Can be modulated by the physical characteristics and motivational significance of stimuli,<br>4. Varies along a continuum that can be quantified in any behavioural state, including wakefulness and low-arousal states including sleep, anaesthesia, and coma,<br>5. Is distinct from motivation and valence but can covary with intensity of motivation and valence,<br>6. May be associated with increased or decreased locomotor activity, and<br>7. Can be regulated by homeostatic drives (e.g., hunger, sleep, thirst, sex).
Behaviour (RDoC)	Affective states, Agitation, Cognition, Emotional Reactivity, Eye Blink, Motivated Behaviour, Motor Activity, Sensory Reactivity, Startle, Waking

How important do you think **Arousal** is to optimal performance in dynamic and high-pressure environments?

- Extremely important
- Very important

- Moderately important
- Slightly important
- Not important / NA
- Don't know / Unsure

Feel free to provide your rationale or reasoning for rating **Arousal** this way.
Please note that your comments here will be read by the research team and may be presented to all Delphi Panel members unless you select 'HIDDEN' in the following question (in which case only the research team will see the comments). For this reason, please ensure your comments are clear and use language that people outside of your field will understand. You will remain anonymous.

OPTIONAL: Please select 'HIDDEN' if you would like your above response to not be included in the anonymous pool of expert comments at the end of the iteration.

CONSTRUCT	Circadian Rhythms
Description (RDoC)	Circadian Rhythms are endogenous self-sustaining oscillations that organize the timing of biological systems to optimize physiology and behaviour, and health. Circadian Rhythms:<br>1. Are synchronized by recurring environmental cues;<br>2. Anticipate the external environment;<br>3. Allow effective response to challenges and opportunities in the physical and social environment;<br>4. Modulate homeostasis within the brain and other (central/peripheral) systems, tissues and organs;<br>5. Are evident across levels of organization including molecules, cells, circuits, systems, organisms, and social systems.
Behaviour (RDoC)	Drive-regulated behaviours, Locomotor activity, Masking, Neurobehavioral functions, Sleep-rated and waking behaviours, Sleep-wake

How important do you think **Circadian Rhythms** is to optimal performance in dynamic and high-pressure environments?

- Extremely important
- Very important
- Moderately important
- Slightly important

- Not important / NA
- Don't know / Unsure

Feel free to provide your rationale or reasoning for rating **Circadian Rhythms** this way.
Please note that your comments here will be read by the research team and may be presented to all Delphi Panel members unless you select 'HIDDEN' in the following question (in which case only the research team will see the comments). For this reason, please ensure your comments are clear and use language that people outside of your field will understand. You will remain anonymous.

OPTIONAL: Please select 'HIDDEN' if you would like your above response to not be included in the anonymous pool of expert comments at the end of the iteration.

CONSTRUCT	Sleep-Wakefulness
Description (RDoC)	Sleep and wakefulness are endogenous, recurring, behavioural states that reflect coordinated changes in the dynamic functional organization of the brain and that optimize physiology, behaviour, and health. Homeostatic and circadian processes regulate the propensity for wakefulness and sleep. Sleep:<br>1. Is reversible, typically characterized by postural recumbence, behavioural quiescence, and reduced responsiveness;<br>2. Has a complex architecture with predictable cycling of NREM/REM states or their developmental equivalents. NREM and REM sleep have distinct neural substrates (circuitry, transmitters, modulators) and EEG oscillatory properties<br>3. Intensity and duration is affected by homeostatic regulation;<br>4. Is affected by experiences during wakefulness;<br>5. Is evident at cellular, circuit, and system levels;<br>6. Has restorative and transformative effects that optimize neurobehavioral functions during wakefulness.
Behaviour (RDoC)	Co-sleeping, Intermediate/ admixed sleep-wake states, Motor behaviours during sleep, Rest-activity patterns, Sensory arousal threshold, Sex-specific sleep behaviours, Sleep, Sleep deprivation and satiation, Sleep inertia, Sleep timing and variability, Sleep-dependent neurobehavioral functions, Wakefulness

How important do you think **Sleep Wakefulness** is to optimal performance in dynamic and high-pressure environments?

- Extremely important
- Very important

- Moderately important
- Slightly important
- Not important / NA
- Don't know / Unsure

Feel free to provide your rationale or reasoning for rating **Sleep Wakefulness** this way.
Please note that your comments here will be read by the research team and may be presented to all Delphi Panel members unless you select 'HIDDEN' in the following question (in which case only the research team will see the comments). For this reason, please ensure your comments are clear and use language that people outside of your field will understand. You will remain anonymous.

OPTIONAL: Please select 'HIDDEN' if you would like your above response to not be included in the anonymous pool of expert comments at the end of the iteration.

Importance: Sensorimotor Systems

DOMAIN: Sensorimotor Systems

Description: Sensorimotor Systems are primarily responsible for the control and execution of motor behaviors, and their refinement during learning and development.

Constructs/Subconstructs

- Construct: Motor Actions
  - Subconstruct: Action Planning and Selection
  - Subconstruct: Sensorimotor Dynamics
  - Subconstruct: Initiation
  - Subconstruct: Execution
  - Subconstruct: Inhibition and Termination
- Construct: Agency and Ownership
- Construct: Habit - Sensorimotor
- Construct: Innate Motor Patterns

[Definitions of key terms.pdf]

The following questions ask you to rate the Subconstructs under the Construct **Motor Actions**

DOMAIN	Sensorimotor Systems
**CONSTRUCT**	**Motor Actions**
Description (RDoC)	A multifaceted construct comprising the processes that must be engaged during the planning and execution of a motor action in a context-appropriate manner. Component processes include action planning and selection, sensorimotor dynamics, initiation, execution, and inhibition and termination. Of note, these processes will often be recruited in conjunction with motivational processes described in other domains, as when appetitive motivations drive approach behaviours. This construct explicitly includes the modulation and refinement of actions during development and learning. The list of subconstructs is not intended to imply a specific order or sequence.
Subconstructs	• Action Planning and Selection<br>• Sensorimotor Dynamics<br>• Initiation<br>• Execution<br>• Inhibition and Termination

CONSTRUCT	Motor Actions
**SUB-CONSTRUCT**	**Action Planning and Selection**
Description (RDoC)	Processes whereby an individual engages a plan for spatial and temporal components of possible purposeful movements, which match internal and external constraints to achieve a goal. This may also include cost-benefit calculations in the development and selection of motor plans.
Behaviour (RDoC)	Conceptual Apraxia, Ideational Apraxia, Ideomotor Apraxia, Limb-Kinetic Apraxia

How important do you think **Action Planning and Selection** is to optimal performance in dynamic and high-pressure environments?

- Extremely important
- Very important
- Moderately important
- Slightly important
- Not important / NA
- Don't know / Unsure

Feel free to provide your rationale or reasoning for rating **Action Planning and Selection** this way.

Please note that your comments here will be read by the research team and may be presented to all Delphi Panel members unless you select 'HIDDEN' in the following question (in which case only the research team will see the comments). For this reason, please ensure your comments are clear and use language that people outside of your field will understand. You will remain anonymous.

OPTIONAL: Please select 'HIDDEN' if you would like your above response to not be included in the anonymous pool of expert comments at the end of the iteration.

CONSTRUCT	Motor Actions
**SUB-CONSTRUCT**	**Sensorimotor Dynamics**
Description (RDoC)	Processes involved in the specification/parameterization of an action plan and program based on integration of internal or external information, such as sensations and urges and modelling of body dynamics. This process is continuously and iteratively refined via sensory information and reward-reinforced information.
Behaviour (RDoC)	Developmental Coordinate Disorder, Hyposensitivity, Weakness

How important do you think **Sensorimotor Dynamics** is to optimal performance in dynamic and high-pressure environments?

- Extremely important
- Very important
- Moderately important
- Slightly important
- Not important / NA
- Don't know / Unsure

Feel free to provide your rationale or reasoning for rating **Sensorimotor Dynamics** this way.

Please note that your comments here will be read by the research team and may be presented to all Delphi Panel members unless you select 'HIDDEN' in the following question (in which case only the research team will see the comments). For this reason, please ensure your comments are clear and use language that people outside of your field will understand. You will remain anonymous.

OPTIONAL: Please select 'HIDDEN' if you would like your above response to not be included in the anonymous pool of expert comments at the end of the iteration.

CONSTRUCT	Motor Actions
**SUB-CONSTRUCT**	**Initiation**
Description (RDoC)	Processes involved in the initiation of a selected action plan; this may include timing of movement onset.
Behaviour (RDoC)	Apathy, Catatonic Stupor, Negative Symptoms, Psychomotor retardation, Stuttering

How important do you think **Initiation** is to optimal performance in dynamic and high-pressure environments?

- Extremely important
- Very important
- Moderately important
- Slightly important
- Not important / NA
- Don't know / Unsure

Feel free to provide your rationale or reasoning for rating **Initiation** this way.
Please note that your comments here will be read by the research team and may be presented to all Delphi Panel members unless you select 'HIDDEN' in the following question (in which case only the research team will see the comments). For this reason, please ensure your comments are clear and use language that people outside of your field will understand. You will remain anonymous.

OPTIONAL: Please select 'HIDDEN' if you would like your above response to not be included in the anonymous pool of expert comments at the end of the iteration.

CONSTRUCT	Motor Actions
**SUB-CONSTRUCT**	**Execution**
Description (RDoC)	Processes involved in the actualization and adaptation of the action implementation.
Behaviour (RDoC)	Activity Level, Ehler Danlos S, Psychomotor retardation

How important do you think **Execution** is to optimal performance in dynamic and high-pressure environments?

- Extremely important
- Very important
- Moderately important
- Slightly important
- Not important / NA
- Don't know / Unsure

Feel free to provide your rationale or reasoning for rating **Execution** this way.

Please note that your comments here will be read by the research team and may be presented to all Delphi Panel members unless you select 'HIDDEN' in the following question (in which case only the research team will see the comments). For this reason, please ensure your comments are clear and use language that people outside of your field will understand. You will remain anonymous.

OPTIONAL: Please select 'HIDDEN' if you would like your above response to not be included in the anonymous pool of expert comments at the end of the iteration.

CONSTRUCT	Motor Actions
**SUB-CONSTRUCT**	**Inhibition and Termination**
Description (RDoC)	Processes involved in the inhibition of motor plans, either before or after an action is initiated, and the sense that a motor plan has been successfully completed. The inhibition sub-construct is commonly operationalized as motor response inhibition and has conceptual overlaps with the Inhibition/Suppression subconstruct of the Cognitive Control construct within the Cognitive Systems domain.
Behaviour (RDoC)	Activity Level, Automatic Obedience, Catatonic Immobility, Catatonic Rituals, Negativism, Perseveration, Stereotypic behaviours, Tics, Utilization Behaviour

How important do you think **Inhibition and Termination** is to optimal performance in dynamic and high-pressure environments?

- Extremely important
- Very important
- Moderately important
- Slightly important
- Not important / NA
- Don't know / Unsure

Feel free to provide your rationale or reasoning for rating **Initiation and Termination** this way.
Please note that your comments here will be read by the research team and may be presented to all Delphi Panel members unless you select 'HIDDEN' in the following question (in which case only the research team will see the comments). For this reason, please ensure your comments are clear and use language that people outside of your field will understand. You will remain anonymous.

OPTIONAL: Please select 'HIDDEN' if you would like your above response to not be included in the anonymous pool of expert comments at the end of the iteration.

CONSTRUCT	Agency and Ownership
Description (RDoC)	The sense that one is initiating, executing, and in control of one's volitional actions and their sensory consequences and the sense that one's body or body parts belong to oneself. This may include the comparison of the predicted and actual sensory consequences of one's action, awareness of the intention to move, temporal binding of self-generated action and their immediate effects, and attenuation of sensory consequences of self-generated actions.
Behaviour (RDoC)	Alien Hand Syndrome, Functional Movement Disorders, Neglect, Perceptions of External Control, Stereotypic behaviours, Tics

How important do you think **Agency and Ownership** is to optimal performance in dynamic and high-pressure environments?

- Extremely important
- Very important

- Moderately important
- Slightly important
- Not important / NA
- Don't know / Unsure

Feel free to provide your rationale or reasoning for rating **Agency and Ownership** this way.
Please note that your comments here will be read by the research team and may be presented to all Delphi Panel members unless you select 'HIDDEN' in the following question (in which case only the research team will see the comments). For this reason, please ensure your comments are clear and use language that people outside of your field will understand. You will remain anonymous.

OPTIONAL: Please select 'HIDDEN' if you would like your above response to not be included in the anonymous pool of expert comments at the end of the iteration.

CONSTRUCT	Habit - Sensorimotor
Description (RDoC)	Learned stimulus-response mappings triggered by internal or external stimuli that are autonomous of the current value of the outcome or goal. Habits may include overlearned sequences. Habits are implicit and efficient, requiring few cognitive resources, but can also be maladaptive under novel circumstances. Habits are based on previous positively or negatively reinforced learning and commonly occur after extended learning. Both habit formation and expression are commonly operationalized within motor control systems. When habit formation is motivated by reward learning it overlaps with the Habit construct within the Positive Valence domain.
Behaviour (RDoC)	Compulsive behaviours, Stereotypic behaviours

How important do you think **Habit - Sensorimotor** is to optimal performance in dynamic and high-pressure environments?

- Extremely important
- Very important
- Moderately important
- Slightly important
- Not important / NA
- Don't know / Unsure

Feel free to provide your rationale or reasoning for rating **Habit - Sensorimotor** this way.
Please note that your comments here will be read by the research team and may be presented to all Delphi Panel members unless you select 'HIDDEN' in the following question (in which case only the research team will see the comments). For this reason, please ensure your comments are clear and use language that people outside of your field will understand. You will remain anonymous.

OPTIONAL: Please select 'HIDDEN' if you would like your above response to not be included in the anonymous pool of expert comments at the end of the iteration.

CONSTRUCT	Innate Motor Patterns
Description (RDoC)	Unlearned action plans that may be triggered by internal or external stimuli. This can include such behaviours as stereotyped expressions of affect, orientation to salience, innate approach and withdrawal phenomena, and startle responses.
Behaviour (RDoC)	Disinhibition of early motor reflexes, Incontinent Affect, Startle, Stereotypic behaviours

How important do you think **Innate Motor Patterns** is to optimal performance in dynamic and high-pressure environments?

- Extremely important
- Very important
- Moderately important
- Slightly important
- Not important / NA
- Don't know / Unsure

Feel free to provide your rationale or reasoning for rating **Innate Motor Patterns** this way.
Please note that your comments here will be read by the research team and may be presented to all Delphi Panel members unless you select 'HIDDEN' in the following question (in which case only the research team will see the comments). For this reason, please ensure your comments are clear and use language that people outside of your field will understand. You will remain anonymous.

OPTIONAL: Please select 'HIDDEN' if you would like your above response to not be included in the anonymous pool of expert comments at the end of the iteration.

Importance: Expert Suggested Constructs

Expert Suggested Constructs

Constructs:

- Shifting
- Processing Speed
- Discomfort Tolerance

Definitions of key terms.pdf

CONSTRUCT	**Shifting**
Description (Delphi Team)	The ability to flexibly switch back and forth between tasks or mental sets.

How important do you think **Shifting** is to optimal performance in dynamic and high-pressure environments?

- Extremely important
- Very important
- Moderately important
- Slightly important
- Not important / NA
- Don't know / Unsure

Feel free to provide your rationale or reasoning for rating **Shifting** this way.
Please note that your comments here will be read by the research team and may be presented to all Delphi Panel members unless you select 'HIDDEN' in the following question (in which case only the research team will see the

comments). For this reason, please ensure your comments are clear and use language that people outside of your field will understand. You will remain anonymous.

OPTIONAL: Please select 'HIDDEN' if you would like your above response to not be included in the anonymous pool of expert comments at the end of the iteration.

CONSTRUCT	Processing speed
Description (Delphi Team)	The speed with which an individual processes many types of information.

How important do you think **Processing Speed** is to optimal performance in dynamic and high-pressure environments?

- Extremely important
- Very important
- Moderately important
- Slightly important
- Not important / NA
- Don't know / Unsure

Feel free to provide your rationale or reasoning for rating **Processing Speed** this way.

Please note that your comments here will be read by the research team and may be presented to all Delphi Panel members unless you select 'HIDDEN' in the following question (in which case only the research team will see the comments). For this reason, please ensure your comments are clear and use language that people outside of your field will understand. You will remain anonymous.

OPTIONAL: Please select 'HIDDEN' if you would like your above response to not be included in the anonymous pool of expert comments at the end of the iteration.

CONSTRUCT	Discomfort tolerance
Description (Delphi Team)	The ability to sit with uncomfortable emotions, states, and sensations (includes stress, boredom, pain, and other negative affective states).

How important do you think **Discomfort tolerance** is to optimal performance in dynamic and high-pressure environments?

- Extremely important
- Very important
- Moderately important
- Slightly important
- Not important / NA
- Don't know / Unsure

Feel free to provide your rationale or reasoning for rating **Discomfort Tolerance** this way.
Please note that your comments here will be read by the research team and may be presented to all Delphi Panel members unless you select 'HIDDEN' in the following question (in which case only the research team will see the comments). For this reason, please ensure your comments are clear and use language that people outside of your field will understand. You will remain anonymous.

OPTIONAL: Please select 'HIDDEN' if you would like your above response to not be included in the anonymous pool of expert comments at the end of the iteration.

Remove / edit prev suggested construct

EDIT CONSTRUCT EXERCISE SUGGESTIONS

In light of voting on all RDoC constructs, would you like to remove or edit any of your previous suggestions? (See here for the complete list of constructs you have just voted on for a refresher)

	Remove	Edit	No, neither
${q://QID41/ChoiceTextEntryValue/2}	○	○	○

	Remove	Edit	No, neither
${q://QID44/ChoiceTextEntryValue/2}	◯	◯	◯
${q://QID45/ChoiceTextEntryValue/2}	◯	◯	◯
${q://QID46/ChoiceTextEntryValue/2}	◯	◯	◯
${q://QID47/ChoiceTextEntryValue/2}	◯	◯	◯

You selected that you wish to edit ${q://QID41/ChoiceTextEntryValue/2} construct. Please detail your edits in the boxes below.

New name of construct:

New definition / description of suggested construct:

New application to Optimal performance:

You selected that you wish to edit ${q://QID44/ChoiceTextEntryValue/2} construct. Please detail your edits in the boxes below.

New name of construct:

New definition / description of suggested construct:

New application to Optimal performance:

You selected that you wish to edit ${q://QID45/ChoiceTextEntryValue/2} construct. Please detail your edits in the boxes below.

New name of construct:

New definition / description of suggested construct:

New application to Optimal performance:

You selected that you wish to edit ${q://QID46/ChoiceTextEntryValue/2} construct. Please detail your edits in the boxes below.

New name of construct:

New definition / description of suggested construct:

New application to Optimal performance:

You selected that you wish to edit ${q://QID47/ChoiceTextEntryValue/2} construct. Please detail your edits in the boxes below.

New name of construct:	
New definition / description of suggested construct:	
New application to Optimal performance:	

Construct Suggestion 2

INSTRUCTION
SUGGESTING CONSTRUCTS

In light of completing the survey, you may suggest additional constructs.
When deciding on these constructs, please consider;

1. Is the construct multifaceted (can it be broken down into smaller constructs/ building blocks)? If so, list the building block constructs instead and provide comment on the greater latent construct you are considering.

2. If it applies to optimal performance in dynamic and high-pressure environments.

Please provide your best description and reasoning for listing the construct. This will be read by the research team who will synthesise the construct according to your description and either match it to an RDoC construct or consult relevant literature to elect it as an additional construct. If an additional construct, the construct will be included in the second iteration of the Delphi survey. The research team will be in contact with you to confirm interpretation of the construct after results have been collected.

Please also select the tick box according to whether you believe the nominated construct is applicable to individual performance (construct important to the one person) or interactional performance (the construct applies in a setting where another person is involved in some capacity).

See here for the complete list of constructs you have just voted on for a refresher.

Do you have a construct to suggest?

- Yes
- No

Construct Suggestion 1:

Contruct Suggestion 1	
Definition / description of suggested construct	
Application to Optimal performance	

Is this construct applied to optimal performance involving only one person (individual), two or more people (interactional), or can it be applied to both?

- Individual
- Interactional
- Both: Individual and Interactional

Do you have an additional construct to suggest?

- Yes
- No

Construct Suggestion 2:

Contruct Suggestion 2	
Definition / description of suggested construct	
Application to Optimal performance	

Is this construct applied to optimal performance involving only one person (individual), two or more people (interactional), or can it be applied to both?

- Individual
- Interactional
- Both: Individual and Interactional

Do you have an additional construct to suggest?

- Yes

○ No

Construct Suggestion 3:

Contruct Suggestion 3	
Definition / description of suggested construct	
Application to Optimal performance	

Is this construct applied to optimal performance involving only one person (individual), two or more people (interactional), or can it be applied to both?

○ Individual
○ Interactional
○ Both: Individual and Interactional

Do you have an additional construct to suggest?

○ Yes
○ No

Construct Suggestion 4:

Contruct Suggestion 4	
Definition / description of suggested construct	
Application to Optimal performance	

Is this construct applied to optimal performance involving only one person (individual), two or more people (interactional), or can it be applied to both?

○ Individual
○ Interactional
○ Both: Individual and Interactional

Do you have an additional construct to suggest?

- Yes
- No

Construct Suggestion 5:

Contruct Suggestion 5	
Definition / description of suggested construct	
Application to Optimal performance	

Is this construct applied to optimal performance involving only one person (individual), two or more people (interactional), or can it be applied to both?

- Individual
- Interactional
- Both: Individual and Interactional

Review

Would you like to review all of your ratings?

- Yes
- No, I'd like to submit all

Powered by Qualtrics
